# C/EBP homologous protein deficiency enhances hematopoietic stem cell function via reducing ATF3/ROS‐induced cell apoptosis

**DOI:** 10.1111/acel.13382

**Published:** 2021-06-15

**Authors:** Zhencan Shi, Daojun Diao, Yanan Zhao, Ying Luo, Yafei Li, Dingdong Liu, Kai Zhang, Yugang Qiu, Li Yu, Zhangfa Song, Zhenyu Ju

**Affiliations:** ^1^ Key Laboratory of Regenerative Medicine of Ministry of Education Institute of Aging and Regenerative Medicine Jinan University Guangzhou China; ^2^ School of Rehabilitation Medicine Weifang Medical University Weifang China; ^3^ Department of Colorectal Surgery Sir Run Run Shaw Hospital Zhejiang University Hangzhou China

**Keywords:** apoptosis, ATF3, C/EBP homologous protein, hematopoietic stem cell function, ROS

## Abstract

Hematopoietic stem cells (HSCs) reside in a quiescent niche to reserve their capacity of self‐renewal. Upon hematopoietic injuries, HSCs enter the cell cycle and encounter protein homeostasis problems caused by accumulation of misfolded proteins. However, the mechanism by which protein homeostasis influences HSC function and maintenance remains poorly understood. Here, we show that C/EBP homologous protein (CHOP), demonstrated previously to induces cell death upon unfolded protein response (UPR), plays an important role in HSCs regeneration. CHOP^−/−^ mice showed normal hematopoietic stem and progenitor cell frequencies in steady state. However, when treated with 5‐FU, CHOP deficiency resulted in higher survival rates, associated with an increased number of HSCs and reduced level of apoptosis. In serial competitive transplantation experiments, CHOP^−/−^ HSCs showed a dramatic enhancement of repopulation ability and a reduction of protein aggresomes. Mechanistically, CHOP deletion causes reduced ATF3 expression and further leads to decreased protein aggregation and ROS. In addition, CHOP^−/−^ HSCs exhibited an increased resistance to IR‐induced DNA damage and improved HSCs homeostasis and function in telomere dysfunctional (G3*Terc* ^−/−^) mice. In summary, these findings disclose a new role of CHOP in the regulation of the HSCs function and homeostasis through reducing ATF3 and ROS signaling.

## Graphical abstract

5‐FU administration and hematopoietic stem cells (HSCs) transplatation induce high rate of protein synthesis and protein aggregation, which further cause impaired HSCs function. Under IR iradiation and telomere‐shortening induced DNA damage, C/EBP homologous protein (CHOP) deficiency protects HSCs from impairment. C/EBP homologous protein deletion reduces ATF3 expression, protein synthesis and rescues HSCs function through reduced ROS and cell apoptosis.

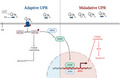

**Abbreviations**
ATF3: activating transcription factor 3
ATF4: activating transcription factor 4
BM: bone marrow
ChIP‐seq: chromatin immunoprecipitation sequencing
CHOP: C/EBP homologous protein
CLP: common lymphoid progenitor
DAPI: 4’,6‐diamidino‐2‐phenylindole
ER stress: endoplasmic reticulum stress
5‐FU: 5‐fluorouracil
HSCs: hematopoietic stem cells
HSPC: Hematopoietic stem and progenitor cell
IR: ionizing radiation
LT‐HSCs: long‐term hematopoietic stem cells
MPP: Multipotent progenitor
PB: peripheral blood
ROS: reactive oxygen species
ST‐HSCs: short‐term hematopoietic stem cells
UPR: unfolded protein response

## 1 INTRODUCTION

Hematopoietic stem cells (HSCs) are subset of cells, reside in bone marrow (BM) niches and have a unique capacity to serve as the origin of life‐long multi‐lineage hematopoiesis (Laurenti & Gottgens, 2018; Morrison & Spradling, 2008; Orkin & Zon, 2008; Scadden, 2006). In the adult BM, HSCs are predominantly maintained in a quiescence state, protecting them from accumulating damages (e.g., DNA damages) and allowing them to support blood production for an entire lifespan (Morrison & Spradling, 2008). In response to hematological stress, such as 5‐FU administration, BM transplantation, and IR radiation, these cells can be readily activated, enter the cell cycle, and robustly repopulate the entire hematopoietic system via multi‐lineage differentiation and self‐renewal, which is accompanied with a high protein synthesis rate (Chandel et al., 2016; Heijmans et al., 2013; Kohli & Passegue, 2014; Laurenti & Gottgens, 2018). Higher protein synthesis upon enhanced cell proliferation and abundant protein production/secretion leads to accumulation of unfolded and misfolded proteins, which is a major cause for ER stress (Luo & Lee, 2013).

Several proteostatic mechanisms in HSCs ensure the integrity of the proteome by promoting efficient folding of unfolded and misfolded proteins, most notably the UPR (UPR^ER^). Prevention of UPR^ER^‐induced apoptosis by the RNA binding protein Dppa5, chemical chaperones, and hypoxia‐inducible factor 2a (HIF2a) limit ER stress and promote HSC function (Miharada et al., 2014; Rouault‐Pierre et al., 2013). In particular, HIF2a limits ROS production in quiescent HSCs, thereby preventing ROS‐induced ER stress, but is dispensable for HSC maintenance (Rouault‐Pierre et al., 2013). Another chemical chaperones, bile acids mitigate ER stress to ensure that rapidly expanding fetal liver HSCs adequately adapt to higher rates of protein synthesis (Sigurdsson et al., 2016). In addition, human HSCs are predisposed to apoptosis by strong activation of the PERK branch of UPR after ER stress, whereas closely related progenitors exhibit an adaptive response leading to their survival (van Galen et al., 2014). In this case, deletion of C/EBP homologous protein (CHOP), a key transcription factor downstream of PERK, prevents cell death upon tunicamycin‐induced unfolded protein response (UPR). However, it is still unknown whether CHOP plays an important role in HSCs regeneration, especially during serial transplantation, DNA damage responses, or the process of aging.

To this end, we used CHOP‐deficient (CHOP^−/−^) mice to explore the functional impact of CHOP on HSCs during homeostasis maintenance, stress response, and aging. In young mice, CHOP^−/−^ HSCs exhibited an increased self‐renewal and differentiation during competitive serial transplantation. C/EBP homologous protein deficiency caused HSCs to recover faster after 5‐fluorouracil (5‐FU) administration due to less apoptosis. For the mechanism, CHOP deletion causes reduced ATF3 expression and decreased protein aggregation, which further reduces ROS and cell apoptosis. In addition, CHOP deletion promoted HSC survival after ionizing radiation (IR) and improved the HSC self‐renewal capacity in mice with short telomeres. Taken together, these data revealed an essential role of CHOP in HSC function and regeneration via ATF3/ROS pathway.

## 2 RESULTS

### 2.1 Lack of CHOP improves hematopoietic stem cell function through reduced apoptosis

A recent study demonstrated that CHOP deficiency shows normal homeostasis of HSC and that CHOP expression increased after cord blood transplantation (van Galen et al., 2014). In line with this study, we found similar results in the analyses of our CHOP^−/−^ mice (Figure S1b–f); and interestingly a 2.5‐fold higher mRNA level of CHOP after transplantation (Figure S2a).

**FIGURE 1:**
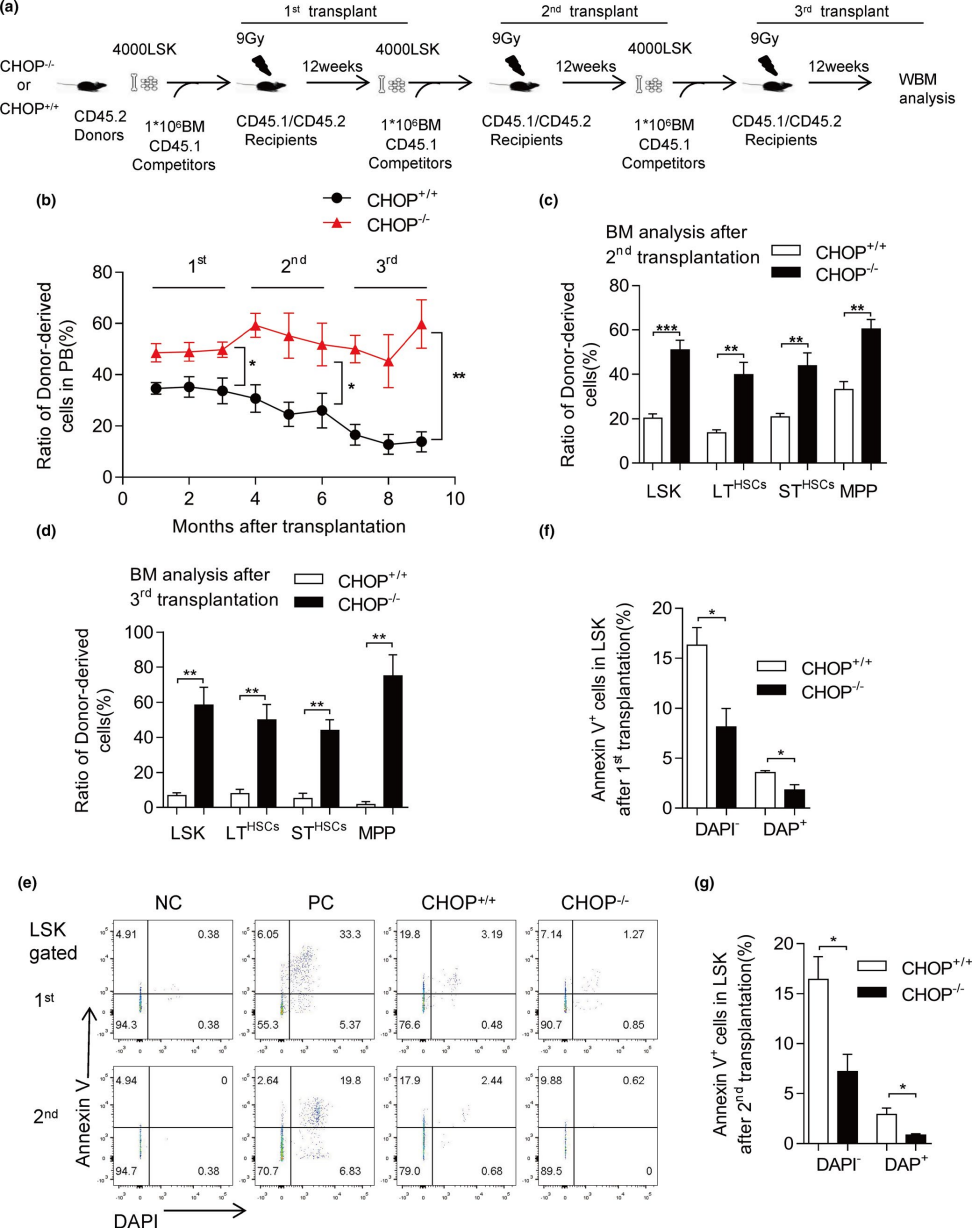
CHOP deficiency improves stem cell function by decreasing apoptosis after transplantation. (a) Experimental schematic for serial competitive transplantation with CHOP^+/+^ or CHOP^−/−^ LSK cells (results in b–d). (b) Three‐round serial transplantation was conducted using 4000 purified LSK cells along with 1 × 10^6^ fresh competitors. Percentage of donor‐derived PB cells at the indicated time points in serial competitive transplantation assay is shown (1st, for the first competitive transplantation, *n* = 6 per group; 2nd, for the second competitive transplantation, *n* = 5 per group; 3rd for the third competitive transplantation, *n* = 5 per group). (c) Percentage of donor‐derived LSK (Lin^−^, Sca‐1^+^, c‐Kit^+^) cells, LT (CD34^−^, Flt‐3^−^ LSK) cells, ST (CD34^+^, Flt‐3^−^ LSK) cells and MPP (CD34^+^, Flt‐3^+^ LSK) cells 12 weeks after secondary transplantation is shown (*n* = 5 per group). (d) Percentage of donor‐derived LSK (Lin^−^, Sca‐1^+^,c‐Kit^+^) cells, LT (CD34^−^, Flt‐3^−^ LSK) cells, ST (CD34^+^, Flt‐3^−^ LSK) cells, and MPP (CD34^+^, Flt‐3^+^ LSK) cells 12 weeks after tertiary transplantation is shown (*n* = 5 per group). (e) The representative FACS plots of apoptosis after transplantation are shown. The positive control was treated with 0.2 μM TG for 12 h. (f, g) The apoptosis was detected with Annexin V/DAPI staining in LSK cells after first transplantation and second transplantation 10 days. The early apoptosis (Annexin V^+^/DAPI^−^) and late apoptotic cells (Annexin V^+^/DAPI^+^) are shown (*n* = 3–4 per group). Data are presented as mean ± SEM. *p < 0.05; **p < 0.01; ***p < 0.001; NS, not significant

To evaluate the self‐renewal and differentiation capacity of CHOP^−/−^ and CHOP^+/+^ HSCs in vivo, we performed a three‐round competitive serial transplantation experiment (Figure 1a). Four thousand LSK cells from young CHOP^−/−^ or CHOP^+/+^ mice (CD45.2) were mixed with 1 × 10^6^ competitor BM cells (CD45.1) and injected into lethally irradiated recipient mice (CD45.1/2). Three months after transplantation, 4000 donor‐derived LSK cells were isolated, mixed with fresh competitor cells, and re‐transplanted into recipient mice. The chimerism in peripheral blood (PB) was examined monthly after transplantation (Figure 1b). CHOP^−/−^ LSK cells showed a higher contribution in the first recipients than the CHOP^+/+^ LSK cells (Figure 1b). Consistently, CHOP^−/−^ BM chimerism showed a threefold increase compared with CHOP^+/+^ BM chimerism in the secondary transplantation (Figure 1c); further, in tertiary recipients, CHOP^−/−^ BM chimerism showed a fivefold increase compared with CHOP^+/+^ BM chimerism (Figure 1d). Further analysis of BM in recipient mice revealed that CHOP deficiency resulted in a significant increase of LT‐HSCs after the second and third transplantation, suggesting that CHOP^−/−^ HSCs have enhanced self‐renewal ability (Figure 1c,d). The chimerism in T lymphocyte, B lymphocyte, and Myeloid cells also showed a clear increase (Figure S2b–d). These data indicate that CHOP^−/−^ HSCs had an enhanced ability to reconstitute the PB after the first transplantation, therefore, CHOP deletion enhanced reconstitution capacity of HSCs in serial transplantation.

To determine the underlying mechanisms, we performed a PY/Hoechst assay to examine the cell cycle state of HSCs after transplantation. However, there was no significant difference in proliferation in HSCs between CHOP^−/−^ and CHOP^+/+^ mice (Figure S2f,g). C/EBP homologous protein is involved in ER stress‐induced apoptosis, which prompted us to examine the ratio of Annexin V^+^ cells in CHOP^−/−^ and CHOP^+/+^ HSCs. After transplantation 10 days, CHOP^−/−^ LSK cells showed a remarkable decrease of Annexin V^+^ cells compared with CHOP^+/+^ LSK cells (Figure 1e,f). While the apoptosis in LT‐HSCs (Flt‐3^−^CD34^−^ LSK cells) has no obvious difference (Figure S2e). After secondary transplantation, CHOP^−/−^ LSK cells also have significantly reduced Annexin V^+^ cells compared with CHOP^+/+^ LSK cells (Figure 1e,g). Taken together, these data indicate that CHOP deficiency enhances HSC self‐renewal and differentiation during serial transplantation by decreasing HSCs apoptosis.

### 2.2 CHOP deletion increases hematopoietic stem cell numbers by reducing apoptosis after 5‐FU administration

**FIGURE 2:**
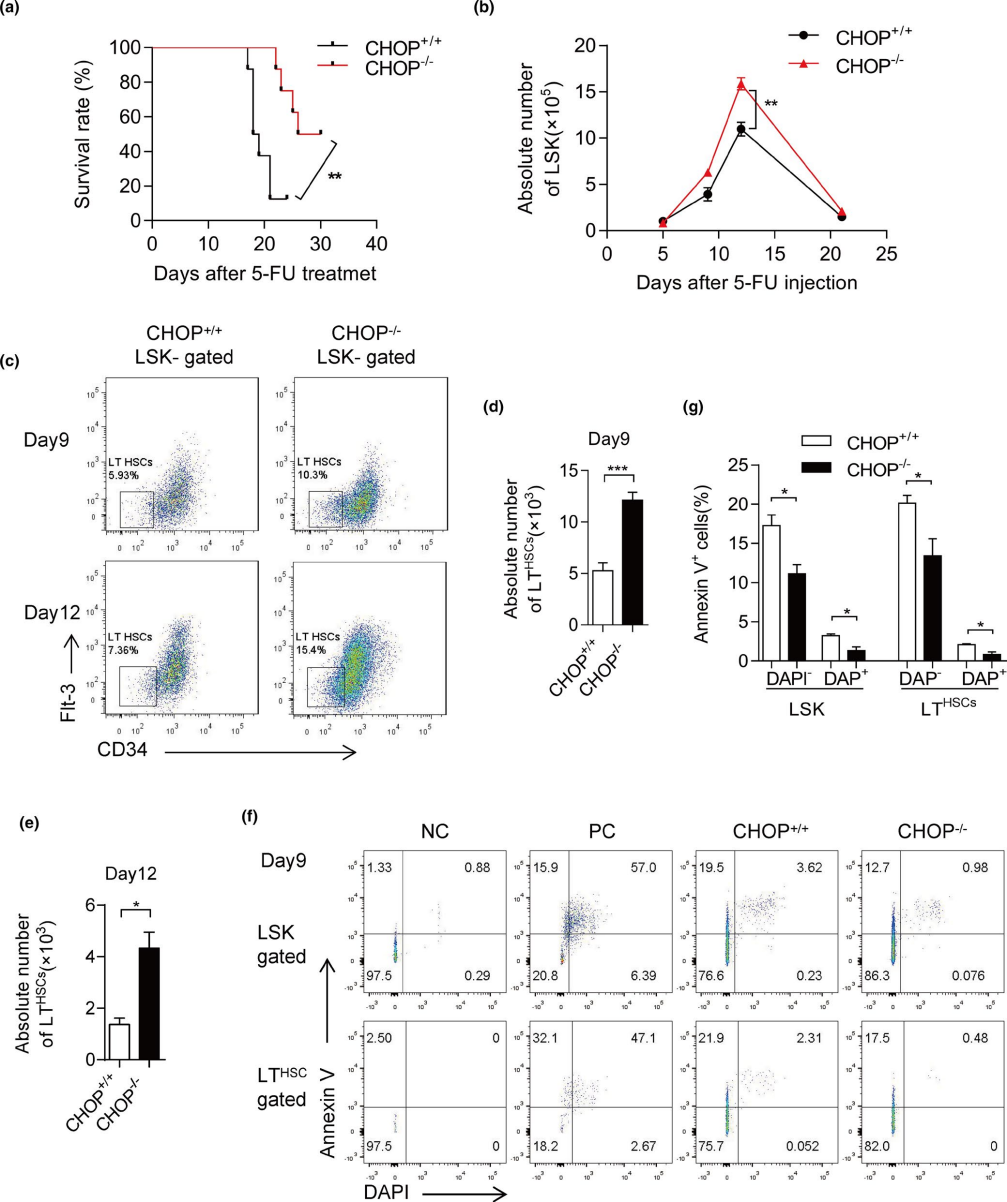
CHOP deletion increases hematopoietic stem cell number via reducing apoptosis after 5‐FU treatment. (a) Survival curve of CHOP^+/+^ and CHOP^−/−^ mice following sequential 5‐FU treatment (*n* = 8 per group). 5‐FU was injected into mice once a week. Representative data from one experiment are shown. (b) Dynamic change of absolute number of LSK cells after 5‐FU treatment detected by flow cytometry (*n* = 3–4 per group). CHOP^+/+^ or CHOP^−/−^ mice were analyzed at the indicated time points after 5‐FU treatment. (c) The representative FACS plots of LT (CD34^−^Flt‐3^−^LSK) cells at day 9 and day 12 after 5‐FU treatment are shown (*n* = 3–4 per group). The frequency of LT cells in LSK cells between CHOP^+/+^ and CHOP^−/−^ mice are shown. (d, e) The absolute numbers of LT cells in BM at day 9 and day 12 after 5‐FU treatment are shown (*n* = 3–4 per group). (f, g) Apoptosis was measured in a donor‐derived population of LSK and LT 9 days after 5‐FU treatment by staining for Annexin V/DAPI. The representative FACS plots are shown on the left (f) and the percentage of Annexin V positive cells is shown on the right (g) (*n* = 3 per group). Data are presented as mean ± SEM. *p < 0.05; **p < 0.01; ***p < 0.001; NS, not significant

To detect the protective effect of CHOP deficiency on hematopoietic stem cells, we challenged CHOP^−/−^ mice with sequential 5‐FU administration. In consequence, CHOP^−/−^ mice died dramatically later than CHOP^+/+^ controls (Figure 2a), indicating that CHOP deficiency protects HSCs from exhaustion caused by 5‐FU administration.

To evaluate the influence of CHOP deletion on the regenerative capacity of HSCs under hematopoietic stress, we analyzed HSCs at different time points following 5‐FU administration (Figure 2b). After administration with 5‐FU, LSK (Lineage^−^, Sca1^+^, c‐kit^+^) cells were drastically depleted in both the CHOP^+/+^ and CHOP^−/−^ mice at day 5 and slightly recovered at day 9 (Figure 2b and Figure S3a). At day 9, the numbers of LT (Flt3‐, CD34‐) cells in CHOP^−/−^ mice were twofold higher than those in CHOP^+/+^ mice; meanwhile, LSK cell numbers in CHOP^−/−^ mice were also significantly higher compared with CHOP^+/+^ litter mates (Figure 2b–d). At day 12, the numbers of both, LSK and LT cells in CHOP^−/−^ mice were significantly higher than those in CHOP^+/+^mice (Figure 2b,c,e). To the end, at day 21, the numbers of LSK cells in CHOP^+/+^ mice both returned to a low level, whereas a higher number of LSK was still present in the CHOP^−/−^ mice (Figure 2b and Figure S3a). These data indicate that CHOP deficiency increased the recovery of HSCs after 5‐FU administration.

After 5‐FU administration, HSCs cells rapidly multiplicated (Figure 2b and Figure S3a), this process may cause an ER stress problem. To examine whether CHOP is involved in this process, we measured the protein level of CHOP at day 9 after 5‐FU administration by fluorescence‐activated cell sorting (FACS) analysis of HSCs and progenitors. Compared to control mice treated with PBS, the protein level of CHOP was significantly increased in progenitors and HSCs (Figure S3b). Real‐time PCR analysis also showed that CHOP mRNA level in LSK cells was higher after 5‐FU administration (Figure S3c), suggesting a modest role of CHOP in regulating the HSCs regenerative process.

In order to find out the underlying mechanisms, we performed a PY/Hoechst assay to examine the cell cycle status of HSCs at day 5, 9, and 12 after 5‐FU administration. There was no significant difference in proliferation in HSCs between CHOP^−/−^ and CHOP^+/+^ mice (Figure S3f–h), while the apoptosis assay showed a remarkable decrease of Annexin V^+^ in CHOP^−/−^ LSK cells compared with CHOP^+/+^ ones after 5‐FU administration 9 days (Figure 2f,g). The ratio of Annexin V^+^ cells in LT cells also decreased significantly in CHOP^−/−^ mice (Figure 2f,g). After 5‐FU administration 5 days, the CHOP^−/−^ LSK cells have reduced Annexin V^+^ cells than CHOP^+/+^ (Figure S3d). While, after 5‐FU administration 12 days, the differences in apoptosis became smaller (Figure S3e).

Together, these data indicate that CHOP deficiency increases HSCs number by decreasing of Annexin V positive cells and therefore promotes survival of HSCs after 5‐FU administration.

### 2.3 CHOP deficiency prevents DNA damage (IR)—induced function decline in HSC

**FIGURE 3:**
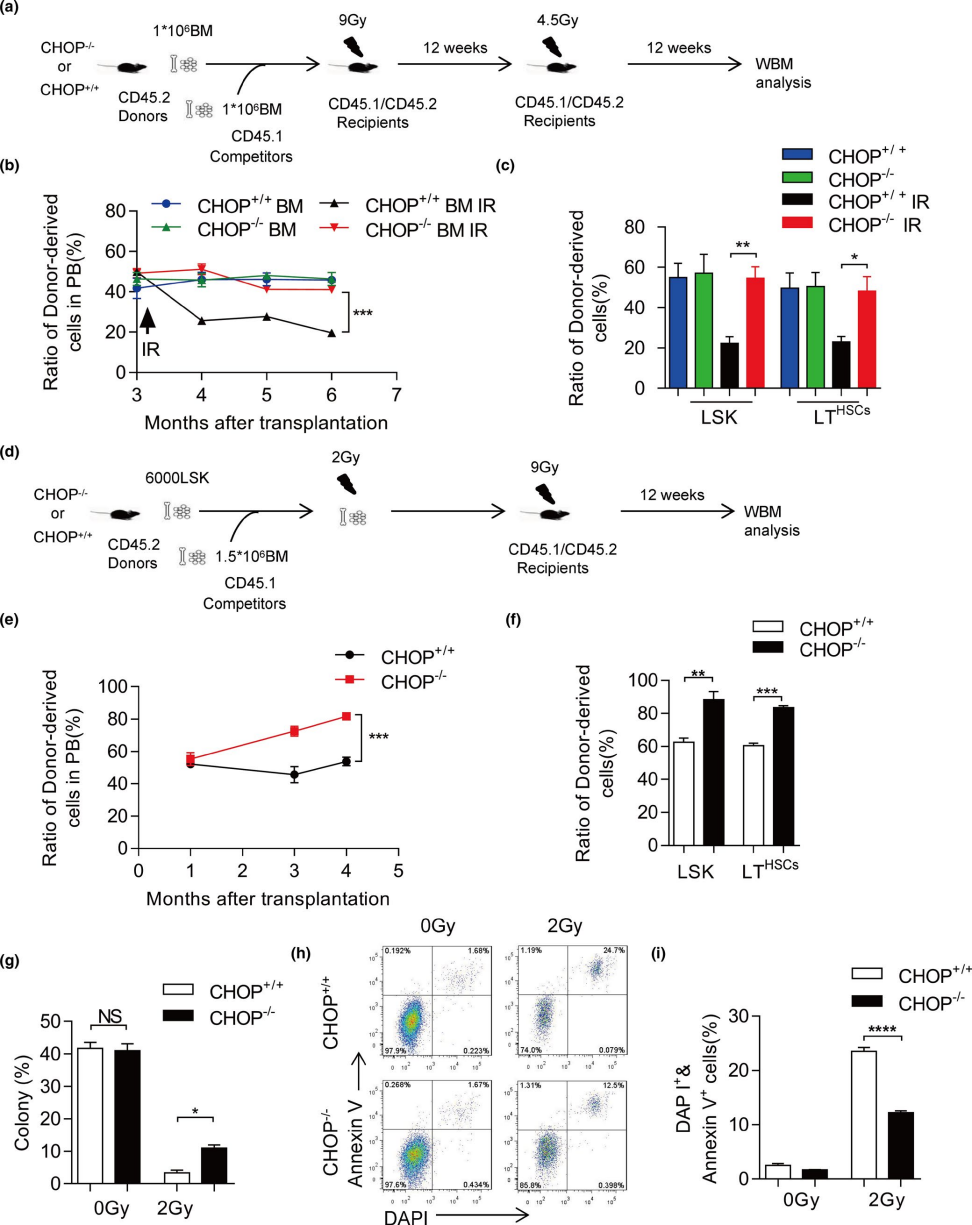
CHOP deficiency ameliorates IR‐induced functional decline in HSCs. (a) Experimental schematic for competitive transplantation with CHOP^−/−^ and CHOP^+/+^ BM cells. After 12 weeks, recipients were irradiated with 4.5‐Gy. (b) 1 × 10^6^ donor BM cells mixed with 1 × 10^6^ competitor BM cells. After 3 months, recipient mice suffered 4.5‐Gy IR. Percentage of donor‐derived PB cells at the indicated time points in BM transplantation after 4.5 Gy IR is shown (*n* = 6 per group). (c) Percentage of donor‐derived LSK (Lin^−^, Sca‐1^+^, c‐kit^+^) cells and LT (CD34^−^, Flt‐3^−^ LSK) cells 12 weeks after BM transplantation after 4.5‐Gy IR is shown (*n* = 5 per group). (d) Experimental schematic for competitive transplantation with CHOP^−/−^ and CHOP^+/+^ LSK, mixed with competitor. Recipients were irradiated with 2‐Gy. (e) 6000 donor LSK cells mixed with 1.5 × 10^6^ competitor BM cells. After in vitro irradiation with 2‐Gy, mixed cells were injected into recipient mice immediately. Percentage of donor‐derived PB cells at the indicated time point in LSK transplantation after 2‐Gy IR is shown (*n* = 3–4 per group). (f) Percentage of donor‐derived LSK (Lin^−^, Sca‐1^+^, c‐kit^+^) cells and LT (CD34^−^, Flt‐3^−^ LSK) cells 16 weeks after LSK transplantation after 2 Gy IR is shown (*n* = 3 per group). (g) Single LT (CD34^−^, Flt‐3^−^ LSK) cells were sorted in 96‐well plates, followed by 0 or 2‐Gy IR, and cultured for 14 days. Colonies were counted in each group (*n* = 3 per group). (h, i) LSK cells were sorted in 48‐well plate and treated with 2‐Gy IR. IR‐induced apoptosis was detected with Annexin V/DAPI staining 16 h after 2‐Gy IR. The representative FACS plots were shown on the left (h), and the percentage of Annexin V positive cells is shown on the right (i) (*n* = 3–4 per group). Data are presented as mean ± SEM. *p < 0.05; **p < 0.01; ***p < 0.001; NS, not significant

Previous studies have shown that CHOP is involved in DNA damage response in MEF cells (Engel et al., 2013). To test whether the effect exists in HSCs in vivo, we performed a BM competitive transplantation experiment (Figure 3a). 1 × 10^6^ BM cells from young CHOP^−/−^ or CHOP^+/+^ mice (CD45.2) were mixed with 1 × 10^6^ competitor BM cells (CD45.1) and injected into lethally irradiated recipient mice (CD45.1/2). The chimerism in PB was examined monthly after transplantation (Figure 3b). Three months after transplantation, we subjected recipient mice (reconstituted with either CHOP^−/−^or CHOP^+/+^ BM cells) to 4.5‐Gy IR. After 4.5‐Gy IR, CHOP deficiency gave rise to enhanced protection to IR, reflected in an increased chimerism in PB (Figure 3b) and BM (Figure 3c). Staining of gamma‐H2AX in CHOP^−/−^ LSK cells and LT‐HSCs (Flt3^−^, CD34^−^) showed decreased levels compared with CHOP^+/+^, suggesting that CHOP deficiency protects HSPCs from DNA damage (Figure S4).

Next, we performed competitive experiment for LSK by IR in vitro (Figure 3d). Six thousand LSK cells from young CHOP^−/−^ or CHOP^+/+^ mice (CD45.2) were mixed with 1.5 × 10^6^ competitor BM cells (CD45.1), after suffering a 2‐Gy IR, then were injected into lethally irradiated recipient mice (CD45.1/2). Unlike the competitive experiment for LSK without IR, the chimerism in PB showed no difference in the first month, however, on the 3rd and 4th month, the chimerism in PB showed significant difference between CHOP^−/−^ and CHOP^+/+^ derived cells (Figure 1b and Figure 3e). After 4 months, CHOP^−/−^ BM chimerism analysis showed a 1.5‐fold increase compared with CHOP^+/+^ BM chimerism (Figure 3f).

To test the effect of CHOP deficiency on the DNA damage response in HSCs, we also conducted a single‐cell colony‐forming assay using HSCs treated with 2‐Gy IR. The suppression of single‐cell colony‐forming ability by IR was significantly less in CHOP^−/−^ HSCs than in CHOP^+/+^ HSCs (Figure 3g), indicating that CHOP deficiency results in a significant IR resistance in HSCs.

To understand the underlying mechanisms, we examined the apoptosis rate by Annexin V/DAPI staining in HSCs 16 h after 2‐Gy IR treatment. Interestingly, at that time point, the induction of apoptosis in CHOP^−/−^ HSCs was less than half of CHOP^+/+^ HSCs (Figure 3h,i). These results indicate that CHOP deficiency also suppresses IR‐induced apoptosis in HSCs.

### 2.4 Loss of CHOP rescues telomere dysfunction‐induced hematopoietic cell function

Telomere dysfunction is another DNA damage‐induced aging stress in HSCs. In line with previous studies, aged mice with dysfunctional telomeres showed that the latter can impair the function and engraftment of hematopoietic stem and progenitor cells, limit B lymphocyte development and increase myeloid proliferation, which are hallmarks of an aging hematopoietic system (Ju et al., 2007). However, whether CHOP plays a role in HSCs of telomere dysfunctional mice is unclear.

**FIGURE 4:**
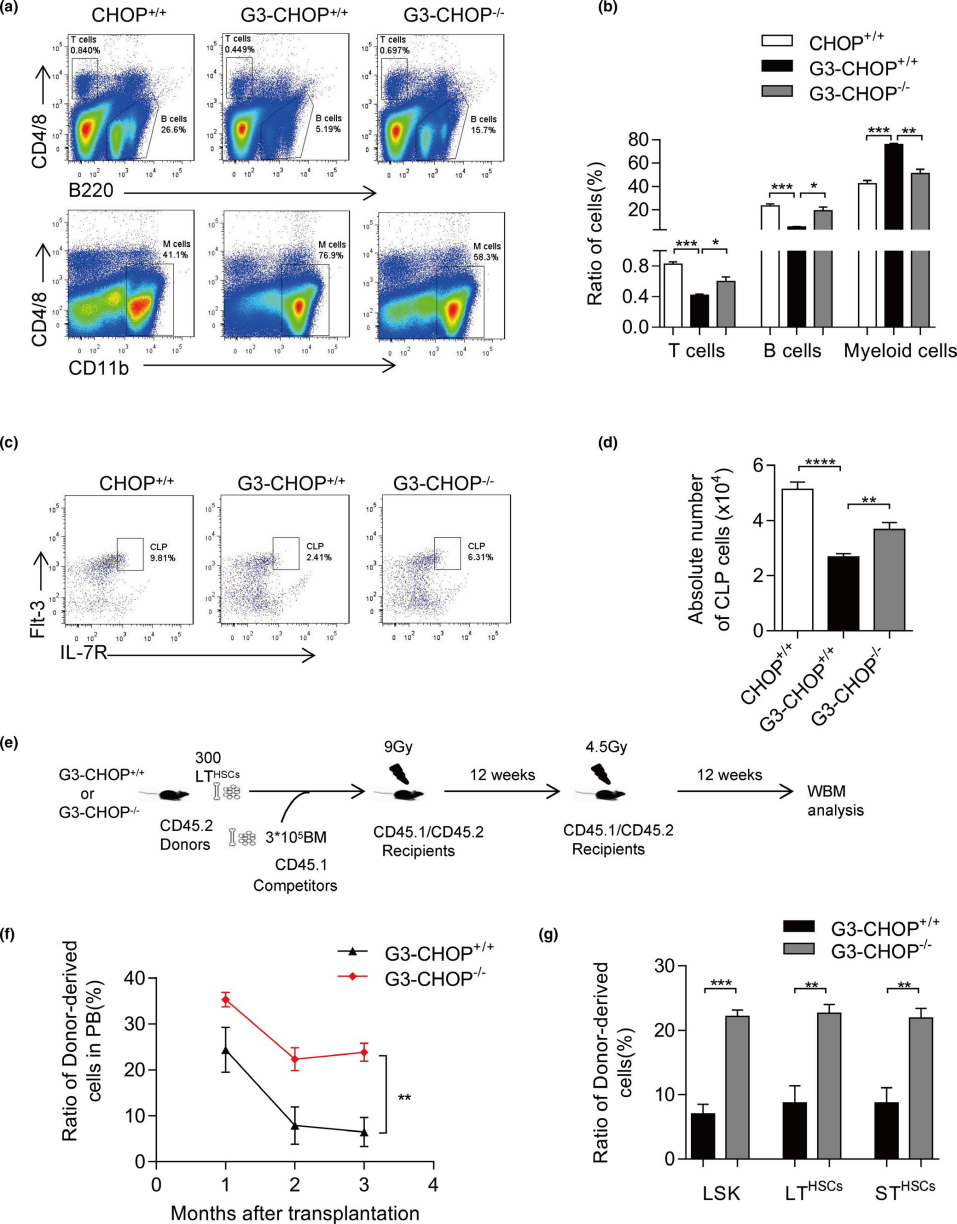
CHOP deficiency improves HSC homeostasis and function in telomere dysfunctional mice. (a) Representative FACS plots of T (CD4/8^+^) cells, B (B220^+^) cells and M (CD11b^+^) cells in the bone marrow in Terc^+/+^, Terc^−/−^, and Terc^−/−^ CHOP^−/−^ mice. (b) Percentage of wild‐type T cells, B cells, and M cells in the bone marrow compared with Terc^−/−^ mice. CHOP deletion improves B cells development and decreases myeloid proliferation (*n* = 3–4 per group). (c) Representative FACS plots of CLP (Lin^−^, Sca‐1^low^, c‐kit^low^, Flt‐3^+^, IL‐7^+^) cells in the bone marrow in Terc^+/+^, Terc^−/−,^ and Terc^−/−^CHOP^−/−^ mice. (d) Absolute number of CLP cells in the bone marrow in Terc^+/+^, Terc^−/−^, CHOP^+/+^and Terc^−/−^ CHOP^−/−^ mice (*n* = 3–5per group). (e) Experimental schematic for competitive transplantation with CHOP^+/+^ or Terc^−/−^ CHOP^+/+^ or Terc‐/‐ CHOP^−/−^ LT cells (results in f and g). (f) The transplantation was conducted using 300 purified LT (CD34^−^, Flt‐3^−^ LSK) cells along with 3 × 10^5^ fresh competitors. Percentage of donor‐derived PB cells at the indicated time points in competitive transplantation assay are shown (*n* = 3–5 per group). (g) Percentage of donor‐derived LSK (Lin^−^, Sca‐1^+^,c‐kit^+^) cells, LT (CD34^−^, Flt‐3^−^ LSK) cells and ST (CD34^+^, Flt‐3^−^ LSK) cells 12 weeks after transplantation are shown (*n* = 3–4 per group). Data are presented as mean ± SEM. *p < 0.05; **p < 0.01; ***p < 0.001; NS, not significant

Here, we crossed Terc^+/−^ and CHOP^+/−^ mice and got the late‐generation of telomerase knockout mice G3CHOP^−/−^ (G3Terc^−/−^CHOP^−/−^) with CHOP deficiency. C/EBP homologous protein deficiency rescued the reduction of B lymphocyte and T lymphocyte ratio in the BM, as well as the reduced B lymphocyte numbers in the spleen of 7‐ to 8‐month‐old G3Terc^−/−^ mice (Figure 4a,b and Figure S5a). In the hematopoietic system, the number of CLP cells (referred to as Sca1^low^ c‐kit^low^ CD127^high^ and Flt3^high^ cells) decreased in G3Terc^−/−^ mice (Figure 4c,d), which could explain the reduction of B lymphocyte and T lymphocyte numbers in the BM; those were also rescued by CHOP deficiency. While the LSK cells and LT‐HSCs (referred to as CD34^−^, Flt3^−^ LSK cells) did not show obvious differences between G3CHOP^+/+^ and G3CHOP^−/−^ mice (Figure S5b).

To evaluate the influence of CHOP deficiency on HSC function of G3Terc^−/−^ mice, we performed a competitive transplantation assay (Figure 4e). 300 LT‐HSCs from G3CHOP^−/−^ or G3CHOP^+/+^ mice (CD45.2) were mixed with 3 × 10^5^ competitor BM cells (CD45.1) and injected into lethally irradiated recipient mice (CD45.1/2). The chimerism in PB was examined monthly after transplantation (Figure 4f). G3CHOP^−/−^ LT cells showed a higher contribution in the first recipients than G3CHOP^+/+^ LT cells (Figure 4f). After 12 weeks, we analyzed the BM of G3CHOP^+/+^ and G3CHOP^−/−^. G3CHOP^−/−^ BM chimerism was fourfold increased compared with G3CHOP^+/+^ BM chimerism (Figure 4g). Furthermore, in the BM, CHOP deficiency decreased the ratio of myeloid cells and increased the ratio of T cells and B cells after transplantation (Figure S5c,d). The chimerism in B lymphocytes and myeloid cells also showed a clear increase (Figure S5e,f).

Together, the above results indicate that CHOP deficiency rescued maintenance of HSCs in G3Terc^−/−^ mice.

### 2.5 CHOP deletion decreases apoptosis by reducing ATF3 expression, protein aggregation, and ROS

C/EBP homologous protein has been reported to induce apoptosis by regulating apoptosis gene expression during ER stress (Kim et al., 2006; Malhotra et al., 2008). However, in HSCs, there was no difference in mRNA level of apoptosis‐related genes between CHOP^−/−^ and CHOP^+/+^ LSK cells at day 3 and 9 after 5‐FU administration (Figure S6a,b).

**FIGURE 5:**
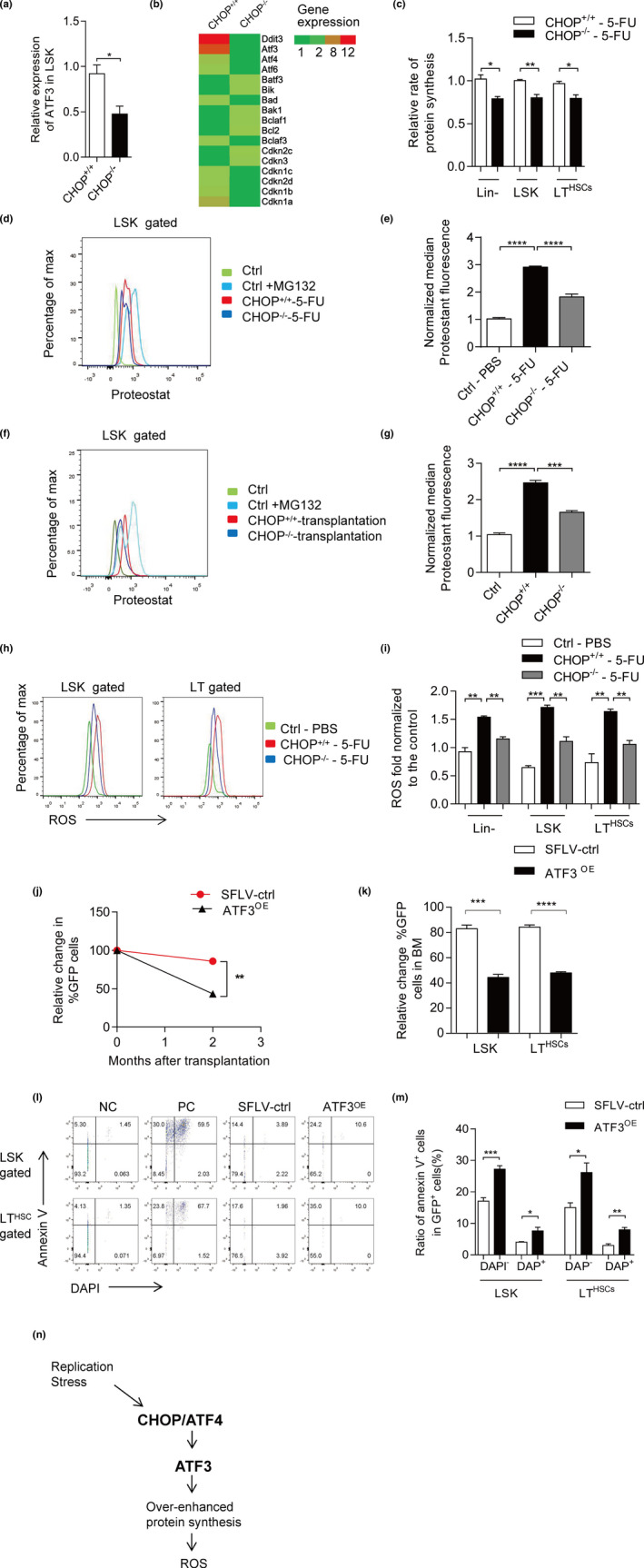
CHOP deletion decreases apoptosis via ATF3/Protein aggregation/ROS axis. (a) The mRNA expression of ATF3 in LSK from CHOP^+/+^ and CHOP^−/−^ mice was measured via real‐time PCR. The relative expression was normalized to β‐actin for statistical analysis (*n* = 3–4 per group). (b) Heat map indicating the relative expression levels of ATF3 and cell cycle‐related gene transcripts in HSCs from indicated genotypes (data are extracted from RNA‐Seq datasets). (c) OP‐Puro fluorescence in Lin^−^ cells, LSK (Lin^−^, Sca‐1^+^, c‐kit^+^) cells and LT (CD34^−^, Flt‐3^−^ LSK) cells 9 days after 5‐FU administration and 1 h after OP‐Puro administration in vivo (*n* = 4 per group). (d, e) The representative FACS plots and Quantification of aggregated and un‐ or misfolded proteins (aggresome) using ProteoStat staining. The change of median Proteostat fluorescence normalized to the median value per replicate to LSK cells at day 9 after 5‐FU treatment is shown (*n* = 4 per group). (f, g) The representative FACS plots and the change of median Proteostat fluorescence normalized to the median value per replicate to the LSK cells after LSK transplantation in vivo are shown (*n* = 3 per group). (h, i) ROS in Lin^−^ cells, LSK (Lin^−^, Sca‐1^+^, c‐kit^+^) cells and LT (CD34^−^, Flt‐3^−^ LSK) cells 9 days after 5‐FU administration. The representative FACS plots are shown (h) and the statistics data are shown below (i) (*n* = 3 per group). (j) ATF3‐cDNA or empty vector infected LSK cells were transplanted along with noninfected cells into lethally irradiated recipients. The infection efficiency was 30% and was normalized to 1. The histogram depicts changes in peripheral blood chimerisms of infected cells (GFP‐positive) in primary recipients at the indicated time points after transplantation (*n* = 3 per group). (k) The histogram depicts changes in bone marrow chimerisms of infected cells (GFP‐positive) in primary recipients at the indicated time points after transplantation (*n* = 3 per group). (l, m) The apoptosis was detected with Annexin V/DAPI staining in LSK and LT‐HSCs GFP^+^ cells after transplantation 10 days. The early apoptosis (Annexin V^+^/DAPI^−^) and late apoptotic cells (Annexin V^+^/DAPI^+^) are shown (*n* = 4 per group). (n) Mechanism for CHOP‐mediated cell apoptosis. In response to replication stress, CHOP was activated, which further induces ATF3 expression. ATF3 contributes to increased GADD34 expression and over‐enhanced protein synthesis. In the end, this process produces ROS as a signal to promote cell apoptosis. Data are presented as mean ± SEM. *p < 0.05; **p < 0.01; ***p < 0.001; NS, not significant

For the underlying mechanism of CHOP in HSCs under stress condition, we applied chromatin immunoprecipitation sequencing (ChIP‐seq) to find out the direct target genes of CHOP in mouse Lin‐ cells. ChIP‐seq analysis identified several CHOP target genes, including ATF3, which was also verified by ChIP‐Q‐PCR (Figure S6e,f). The Q‐PCR results showed specifically that the expression of ATF3 decreased in CHOP^−/−^ LSK cells in compare with CHOP^+/+^ LSK cells, which was also shown in RNA‐seq data (Figure 5a,b and Figure S6c). Increased ATF3 may promote Gadd34 expression, which directs the type 1 protein phosphatase (PP1) to dephosphorylate eIF2 (Jiang et al., 2004) and leads to protein synthesis. Han et al. found that overexpression of CHOP induces apoptosis in MEFs through transcriptionally increasing the protein synthesis‐related genes and ROS (Han et al., 2013). However, the molecular mechanism of CHOP‐induced apoptosis in HSCs seems unclear; therefore, we hypothesize that CHOP may regulate HSC apoptosis through ATF3‐induced protein synthesis and ROS. The protein synthesis rate was checked in HSCs of CHOP^+/+^ and CHOP^−/−^ mice after 5‐FU administration. OP‐Puro assay, an effective method to measure the protein synthesis in the cell, in which the treated mice cells show increased fluorescence level then untreated mice (Signer et al., 2014). After 9 days of 5‐FU administration, OP‐Puro was injected into mice and the mice were sacrificed 1 h later. The result showed that CHOP^−/−^ mice had less protein synthesis than CHOP^+/+^ mice in hematopoietic stem and progenitor cells (Figure 5c).

Furthermore, we checked the level of aggregated proteins via the protein aggregation kit after 9 days of 5‐FU administration (Sigurdsson et al., 2016). The level of aggregated proteins was significantly increased within the HSC population of CHOP^+/+^ mice, while the CHOP^−/−^ mice contained only half the level of aggregated proteins in comparison with CHOP^+/+^ mice (Figure 5d,e). Similarly, after transplantation CHOP^−/−^ mice had less aggregated proteins than CHOP^+/+^ mice (Figure 5f,g).

After 9 days of 5‐FU administration, ROS level increased in hematopoietic stem‐ and progenitor cells. C/EBP homologous protein deficiency rescues the increased ROS level due to 5‐FU significantly (Figure 5h,i). The LT‐HSCs with high level of ROS showed high expression of CHOP (Figure S6h), indicating CHOP plays an important role of it. 5‐FU administration significantly reduced expression of several anti‐oxidative stress response genes, including Sod3, Mt2, and Dusp1, in a CHOP‐dependent way, which may also contribute to increased oxidative stress and reduced survival rate of wild‐type mice (Figure S6d).

Furthermore, the overexpression of ATF3 in CHOP^−/−^ LSK cells showed decreased percentage of GFP‐positive donor‐derived cells in PB and BM (Figure 5j,k and Figure S6g). The apoptosis analysis also showed that overexpression of ATF3 has increased Annexin V^+^ cells compared with SFLV‐ctrl cells (Figure 5l,m).

These data demonstrate that, under stress condition, CHOP induces ATF3 expression, protein synthesis, and ROS level and further causes cell apoptosis. However, those effects were rescued by CHOP deletion.

## 3 DISCUSSION

C/EBP homologous protein has been reported as a transcription factor‐induced downstream of endoplasmic reticulum stress and DNA damage response (Engel et al., 2013; Oyadomari & Mori, 2004; Suzuki et al., 2017), its deletion could inhibit apoptosis according to the context (Doerks et al., 2002; Han et al., 2013). However, the molecular mechanisms that mediate CHOP‐induced apoptosis in HSCs remain a mystery.

The protein level of CHOP remains low on homeostasis; after tunicamycin (TM) treatment for 4 h, CHOP expression increased visibly in wild‐type (WT) mouse embryonic fibroblasts (MEFs) (Han et al., 2013). Previous analysis of CHOP‐deficient BM showed a small increase in the quantity of HSPCs. Our study also showed similarly that CHOP deficiency exhibited a normal phenotype regarding homeostasis (van Galen et al., 2014). After hematological stress, 5‐FU treatment and transplantation, HSC numbers increased, accompanied with a high rate of protein synthesis. A high rate of protein synthesis induced by cells proliferation could lead to aggregates of mis/unfold proteins, which further cause an ER stress response. The acute ER stress‐induced apoptosis occurs while cells rapidly proliferating. Although LSK cells and LT‐HSCs have more early apoptosis (Annexin V+ DAPI−), late apoptosis (Annexin V+ DAPI+) is rare. At the same time, the proliferation rate of LSK cells and LT‐HSCs was significantly higher than the proportion of apoptotic cells; therefore, the cell amount keeps increasing.

Previous study showed that after cord blood transplantation, CHOP and GADD34 expression increased, consistent with a stress response (van Galen et al., 2014). During this process, CHOP is highly expressed and participates in the ER stress‐induced apoptosis (Doerks et al., 2002; Han et al., 2013; Kim et al., 2006; Wielenga et al., 2015). Decreased expression of CHOP rescues the apoptosis and increases the frequency and number of HSCs obviously.

Studies have demonstrated that CHOP induces the expression of apoptosis genes, such as DR5, TRB3, BIM, and PUMA, and represses the expression of BCL2, which triggers apoptosis during ER stress (Kim et al., 2006; Malhotra et al., 2008). In macrophages, CHOP induces the transcription of Ero1α, which then activates calcium‐mediated apoptosis (Signer et al., 2014). Moreover, CHOP interacts and forms heterodimer with ATF4 which initiate the restoration of mRNA translation, leading to increased protein synthesis, ATP depletion, oxidative stress, and cell death in MEFs (Han et al., 2013). However, 5‐FU administration did not lead to decreased expression of apoptosis genes, such as Bid, Bim, and Bcl‐2, whereas displayed a reduced level of aggregated proteins in CHOP^−/−^ mice. Increased accumulation of protein aggregates reduces the ability of quiescent NSCs to be activated during aging (Leeman et al., 2018). Bile acid (BA), as a chemical chaperone, reduces the level of protein aggregates in FL‐HSCs and improves HSCs function after cultured in vitro (Sigurdsson et al., 2016). Increased protein synthesis is accompanied with ROS, which are a necessary signal for apoptosis before restoration of proteostasis (Han et al., 2013). Here, we show that CHOP deficiency reduces the expression of ATF3, which may through inhibiting GADD34 activation, decreases the level of protein aggregates by reducing the rate of protein synthesis in HSCs after 5‐FU treatment and transplantation. Overexpression of ATF3 in CHOP deficiency LSK cells showed decreased chimerism in PB and BM. The ATF3^OE^ LSK cells have induced apoptosis compared with SFLV‐ctrl. Taken together, these results indicate that CHOP deficiency decreases the level of apoptosis by decreasing ATF3 expression, protein aggregation, and oxidative stress.

Interestingly, we found that the percentage of donor‐derived cells in PB shows an obvious increase during the first month, and then remains stable during the next 2 months after first and secondary transplantation, while, during the BM transplantation before 4.5 Gy IR, the chemism in the first 3 months shows no difference (data no shown). The 5‐FU administration assay shows that the number of LSK cells recovered quickly from day 5 to day 9. C/EBP homologous protein‐deficient mice had a 2.5‐fold increased LT cells number, while the number of LSK cells showed no obvious difference. These results indicate two points of information, (1) stress responses mostly happened in fast‐expanding cells; (2) pure HSCs are more sensitive to ER stress response. In support of our finding, TM‐induced ER stress causes more apoptosis in human HSCs compared to progenitors (van Galen et al., 2014).

CHOP, also named ddit3, is involved in DNA damage response through a unique pathway independent of either protein kinase C or tyrosine kinase. Previous study showed that CHOP may indirectly promote neuronal survival by restraining p53 induction via MDM2 upregulation (Engel et al., 2013). Our study showed that CHOP deficiency increases the percentage of donor‐derived cells after IR in vivo and in vitro, implying CHOP deficiency protects HSCs from IR‐induced DNA damage. Unlike normal HSC transplantation, the percentage of donor‐derived cells in the first month did not differ with CHOP^−/−^ HSCs IR‐irradiated with 2 Gy in vitro. This shows that IR‐induced DNA damage plays a more important role in this assay compared to the other stress responses. C/EBP homologous protein‐deficient LSK showed lower level of apoptosis and had an increased clone number after 2 Gy IR in vitro, which indicates an involvement of CHOP in IR‐induced apoptosis and suggests CHOP deficiency protects HSCs from acute DNA damage caused apoptosis in vivo and in vitro.

Previous studies showed that telomere dysfunctional mouse models exhibit an aging phenotypes with impaired HSC maintenance and function (Ju et al., 2007). P21 deficiency rescued proliferation of intestinal progenitor cells and improved the repopulation capacity and self‐renewal of hematopoietic stem cells without rescuing telomere length in telomere dysfunctional mice (Choudhury et al., 2007). Our data showed a decreased B lymphocyte number and myeloid cells expansion in BM and spleen in 7‐ to 8‐month‐old G3Terc^−/−^ mice, while HSCs displayed a threefold increase but had an impaired function. In telomere dysfunctional CHOP‐deficient mice, we found CHOP deficiency can clearly increase B lymphocyte numbers and decrease myeloid numbers in BM and spleen. Transplantation assays further show that CHOP deficiency partly rescues the function of HSCs. While there was no difference of the ATF3 expression in G3CHOP^+/+^ mice and G3CHOP^−/−^ mice (Figure S5g), these results indicate that CHOP deficiency improves HSCs homeostasis and function in telomere dysfunctional mice models, which may not through ATF3 regulating pathways. In G3Terc knockout mice, the expression of p53 is high and CHOP has been reported to regulate p53 (Engel et al., 2013). Therefore, CHOP may regulate p53 to rescue the G3Terc knockout mice phenotype.

The vascular endothelial cells that provide support for HSC are affected by 5‐FU treatment and respond to HSC in a similar manner during recovery or aging (Xie et al., 2009). Megakaryocytes serve as HSC‐derived niche cells to maintain HSC quiescence and promote post‐injury regeneration through TGF‐b–SMAD signaling. CHOP^−/−^ and also Terc^−/−^ mice which have a global knockout in hematopoietic cells (Zhao et al., 2014). CHOP^−/−^ mice may also provide a “rejuvenated” environment supportive of HSC expansion and maintenance after 5‐FU treatment or aging.

Even though CHOP deficiency HSCs expand better and have less accumulation of unfold proteins, while CHOP deficiency has a bad effect in some other tissues and organs. Such as in brain, CHOP promotes neuronal survival from epilepsy by restraining p53 (Engel et al., 2013). C/EBP homologous protein deficiency causes obesity in female mice. Sometimes, evolution is aimed for the better continuation of species, not for individual survival.

In summary, our study provides a novel concept of how CHOP deficiency protects HSCs from replication stress and DNA damage‐induced apoptosis. C/EBP homologous protein deficiency reduces ATF3 expression, protein synthesis rate, and ROS synthesis, which cause CHOP‐mediated apoptosis (Figure 5n). In telomere dysfunctional mice, CHOP deficiency rescued HSCs homeostasis and function. The HSC niche can also be influenced by CHOP^−/−^ and provide a “rejuvenated” environment supportive of HSC expansion and maintenance after 5‐FU treatment or aging. C/EBP homologous protein deficiency improves HSCs function in all kind of stress response through ATF3/ROS axis.

## 4 EXPERIMENTAL PROCEDURE

### 4.1 Mice

CHOP^−/−^ mice were a kind gift from Professor Lili and were maintained in a C56BL/6 (CD45.2) background. CHOP^−/−^ mice were crossed with Terc^+/−^ mice to generate Terc^+/−^ CHOP^+/−^ mice. These mice were crossed through successive generations to produce G3Terc^−/−^ CHOP^−/−^ mice. The recipient mice used in the competitive transplantation assays were either CD45.1 mice or CD45.1/CD45.2 mice. The Animal Care and Ethics Committee at Jinan University approved all animal experiments in our study.

### 4.2 Flow cytometry and cell sorting

Bone marrow cells were incubated in a lineage cocktail containing antibodies against CD4 (1:100, RM4‐5), CD8 (1:100, 53‐6.7), Ter‐119 (1:100, TER‐119), CD11b (1:150, M1/70), Gr‐1 (1:150, RB6‐8C5), and B220 (1:100, RA3‐6B2) for 30 min. After washing, the cells were incubated in CD34 (1:100,RAM34), CD48 (1:200, HM48‐1), CD45.2 (1:100, 104), IL‐7R (1:100, A7R34), Flt3 (1:100, A2F10), CD150 (1:100, TC15‐12F12.2), CD45.1 (1:100, A20), Sca1 (1:100, E13‐161.7), c‐Kit (1:100, ACK2), CD16/32 (1:100, 93), and streptavidin. All monoclonal antibodies were from BD Biosciences. Before cell sorting, first, the BM cells were enriched with anti‐antigen‐presenting cell microbeads (Miltenyi Biotec) and then stained with antibodies for surface markers. Data acquisition and cell sorting were performed using a LSRFortessa (BD Biosciences) cell analyzer or an Influx cell sorter (BD Biosciences). The data were analyzed using the FlowJo software.

### 4.3 Cell culture

For liquid culture, cells were cultured in serum‐free expansion medium (SFEM) (Stem cell Technologies) with 50 ng/ml stem cell factor (Pepro Tech), 50 ng/ml Thrombopoietin (Pepro Tech), and 100 U/ml penicillin/streptomycin.

### 4.4 Single‐cell colony‐forming assay

Single cells were cultured for 14 days in liquid medium supplemented with 10% fetal bovine serum (FBS, Life technologies), 20% BIT 9500 (Stem cell Technologies), 2 mM L‐glutamine (Life technologies), 100 U/ml penicillin/streptomycin, 5 × 10^−5^ M β‐ME (Sigma‐Aldrich), 10 ng/ml stem cell factor (SCF, Pepro Tech), 10 ng/ml thrombopoietin (TPO, Pepro Tech), and 10 ng/ml Interleukin‐3 (IL‐3, Pepro Tech). Three classes of colonies were defined large colonies consisting of more than 10,000 cells, intermediate colonies consisting of more than 1000 cells, small colonies consisting of more than 100 cells.

### 4.5 5‐FU experiment

5‐FU (150 mg/kg) was injected in mice. Mice were euthanized at the indicated time point and subjected to fluorescence‐activated cell sorter (FACS) analysis. Analysis of cell cycle and apoptosis in vivo indicated cells were stained with antibodies (PY/Hoe or Annexin V), followed by FACS analysis.

### 4.6 Transplantation assay

For HSCs transplantation, 4000 LSK cells or 300 CD48^−^CD150^+^ LT cells were FACS‐sorted, mixed with 1 × 10^6^ or 3 × 10^5^ competitor BM cells and transplanted into lethally irradiated mice. For the second and third transplantations, 4000 Donor LSK cells were sorted, mixed with 1 × 10^6^ BM cells and transplanted into the next recipient mice. For the BM transplantation assay, 1 × 10^6^ BM cells from donor mice were injected into lethally irradiated recipient mice with competitor cells.

### 4.7 Analysis of the cell cycle and apoptosis

Cell cycle analysis was performed via PY/Hoechst staining. After surface markers labeling, BM cells were incubated with 5 μg/ml Hoechst 33342 (MedChemExpress) in Hank balanced salt solution containing 20 mM HEPES, 5 mM glucose, and 10% fetal bovine serum at 37°C for 45 min, followed by an additional 45 min of incubation with 1 μg/ml Pyronin Y (Sigma‐Aldrich). Cells were subsequently analyzed on a Fortessa flow cytometer (BD Biosciences). Annexin V Apoptosis Detection Kits (BD Pharmingen) were used for the apoptosis analyses. For the in vitro assays, the cells were sorted and cultured in SFEM (Stemcell Technologies) medium containing (50 ng/ml) SCF and (50 ng/ml) TPO and were analyzed via BrdU incorporation and Annexin V staining assays.

### 4.8 Quantitative reverse transcriptase‐polymerase chain reaction

Total RNA was extracted using the RNeasy Micro Kit (Qiagen) and reverse transcribed using PrimeScript RT Master Mix (TaKaRa Biotechnology). Real‐time PCR for the genes was performed using KAPA SYBR FAST qPCRKits (Kapa Biosystems) on the CFX 96 Real‐time System (Bio‐Rad). The amount of target RNA was normalized to that of the endogenous control β‐actin. The gene expression quantities were determined according to the relative Ct method.

### 4.9 Measurement of protein synthesis

OP‐Puro (50 mg/kg body mass; pH 6.4–6.6 in PBS) was injected intraperitoneally. One hour later mice were sacrificed. Bone marrow was collected, and 1 × 10^7^ cells were stained with combinations of antibodies against cell‐surface markers as described. After washing, the cells were fixed, permeabilized, the azide‐alkyne cycloaddition was performed using the Click‐iT Cell Reaction Buffer Kit (Life Technologies) and azide conjugated to Alexa Fluor 488 at 5 mM final concentration. After the 30 min reaction, the cells were washed twice in PBS supplemented with 3% fetal bovine serum, 0.1% saponin, then resuspended in PBS and analyzed by flow cytometry. “Mean of OP‐Puro fluorescence” reflected absolute fluorescence values for each cell population from multiple independent experiments.

### 4.10 Quantification of aggregated proteins (proteoStat staining)

After 5‐FU administration or transplantation, mice were sacrificed. Bone marrow was collected, and 1 × 10^7^ cells were stained with combinations of antibodies against cell‐surface markers as described. After washed, BM cells were analyzed fresh with ProteoStat staining (Enzo Life Sciences). Cells were fixed in Cytofix/Cytoperm Fixation and Permeabilization Solution (BD) for 30 min. Fixed cells were stained with the ProteoStat dye (1:10,000) dilution in permeabilization solution for 30 min. Cells were washed and analyzed on Fortessa (BD). For positive control, after surface markers labeling, BM cells were cultured in SFEM with proteasome inhibitors MG‐132 for 12 h.

### 4.11 Intracellular staining for ROS analysis

After 5‐FU administration, BM was collected. 1 × 10^7^ cells were stained with combinations of antibodies against cell‐surface markers as described. After washed, cells were fixed with 20 µM DCFDA for 30 min at 37°C in conical test tube. Cells were washed and analyzed on Fortessa (BD).

### 4.12 Intracellular staining of CHOP

After 5‐FU administration, BM was collected. 1 × 10^7^ cells were stained with combinations of antibodies against cell‐surface markers as described. After washed, cells were fixed in Cytofix/Cytoperm Fixation and Permeabilization Solution (BD) for 30 min. Fixed cells were stained with anti‐CHOP (Acris) in permeabilization solution for 30 min. Cells were washed and analyzed on Fortessa (BD).

### 4.13 ChIP‐seq assay

In brief, 10 million FACS‐sorted Lin‐ cells from in vitro BM cultures were cross‐linked for 10 min in 1% formaldehyde (Sigma‐Aldrich) at room temperature. The cross‐linked Lin‐ cells were performed using a commercial kit (EZ‐ChIP Kit; Millipore) according to a published protocol (Wang et al., 2016). The antibody used in CHIP‐seq was CHOP (CST). The ChIP‐seq analysis was done by Guangzhou MAGIGEN Biotechnology Co., Ltd. Briefly, FastQC was used to make quality statistics of the original data and then Trimmomatic was used to prune the original data, remove the joint sequence, and remove the low‐quality base; and the length of the remaining reads after pruning, which is longer than 18nt, was selected for the follow‐up analysis. In the end, the quality of clean reads after pruning is counted by FastQC. The MAPQ value was used to evaluate the comparison results. The probability of non‐unique comparison of reads is only 5% when 13 is chosen as the threshold value. After the quality control of the above sequencing data and the comparison with the reference genome, in order to obtain the binding site information of protein and DNA, MACS2 software was used to perform peak calling analysis and subsequent series of analysis of the peak detection results. MEME and DREME software were used to detect the significant Motif sequence in peak sequence, and then Tomtom software was used to compare the Motif sequence with the known Motif database, and annotate it with the known Motif.

### 4.14 ChIP‐qPCR

ChIP‐qPCR was performed using a commercial kit (EZ‐ChIP Kit; Millipore) according to a published protocol (Wang et al., 2016). The antibodies used in ChIP‐qPCR were as follows: CHOP (CST). The primer sequences of ATF3 ChIP are listed, Forward: TGAGTGAGACTGTGGCTGGGA, Reverse: ATTGGTAACCTG GAGTTA AGCGGG.

### 4.15 Lentivirus production

ATF3 overexpression lentivirus was produced in 293T cells after transfection of 15 mg SFLV‐cDNA‐EGFP plasmid, 9 mg PSPAX2 plasmid, and 4.5 mg PMD.2G plasmid. According to standard procedures, virus was concentrated by centrifugation at 100,000*g* for 2.5 h, 4°C, virus pellet was resuspended in sterile PBS.

### 4.16 Lentivirus infection

Fresh‐isolated CHOP^−/−^ LSK (4000/per recipient mouse) cells were plated in 100 µl serum‐free expansion medium (SFEM; Stem Cell; 09650) with 100 U/ml penicillin, 100 µg/ml streptomycin, 50 ng/ml thrombopoietin (TPO; Peprotech; 315‐14), and 50 ng/ml stem cell factor (SCF; Peprotech; 250‐03) in a 96‐well plate. Lentivirus suspensions were added into cells according to titration results. After 12 h, cultured LSK cells were collected and mixed with competitor mice BM (1 × 10^6^/per mouse) cells, then injected into recipient mice. The surplus infected LSK cells were analyzed after cultured 3 days as initial point.

### 4.17 RNA‐seq assay

RNA‐seq was conducted on WT and CHOP^−/−^ LSK cells. Total RNA of cells was extracted using MagMAX‐96 Total RNA Isolation kit (Ambion; AM1830) according to the manufacturer's protocol. Briefly, RNA samples for transcriptome analysis were pretreated with DNase and processed following Illumina manufacturer's instructions where magnetic beads with oligo (dT) were used to isolate polyadenylated mRNA (polyA+ mRNA) from the total RNA. Fragmentation buffer consisting of divalent cations was added for shearing mRNA to short fragments of 200–700 nucleotides in length. These short fragments were used as templates to synthesize the first‐strand cDNA using random hexamer primer. The second‐strand cDNA was synthesized using buffer including dNTPs, RNase H, and DNA polymerase I. The products were purified and resolved with QIAquick PCR Purification Kit (Qiagen) and Elution buffer for end preparation and tailing A, respectively. Purified cDNA fragments were connected with sequencing adapters and gel electrophoresed to select suitable fragments for PCR amplification. Agilent 2100 Bioanalyzer and Applied Biosystems StepOnePlusTM Real‐Time PCR System were used in quantification and qualification of the sample library for quality control. The sequencing reads were mapped to the mouse reference genome (mm9) using HISAT. Differentially expressed genes (DEGs) between each genotype were calculated by standard bioinformatic analysis package, and the hierarchical clustering for DEGs between samples was generated based on DEGs.

### 4.18 Statistical analysis

Data are presented as mean ± standard Error of Mean. The statistical significance of the differences between groups was calculated using the unpaired Student *t* test, and the survival curve was analyzed using a log‐rank (Mantel‐Cox) test.

## CONFLICTS OF INTEREST

None declared.

## AUTHOR CONTRIBUTIONS

D.D. and Z.J. designed the experiments and supervised the project. Z.S., Y.Z., Y.L., Y.L., D.L., and K.Z. performed the experiments. Z.S. and D.D. analyzed the data and prepared the manuscript and figure; Y.Q., L.Y., and Z.S. provided valuable help. D.D. and Z.J. oversaw the preparation of the manuscript, commented on and revised the manuscript.

## Supporting information

Figure S1

Figure S2

Figure S3

Figure S4

Figure S5

Figure S6

Supplementary Material

## Data Availability

The data that support the findings of this study are available from the corresponding author upon reasonable request (https://data.mendeley.com/drafts/cnv5p5rszz).
