# Supplementary figures and images for: C/EBP homologous protein deficiency enhances hematopoietic stem cell function via reducing ATF3/ROS‐induced cell apoptosis
Source: Aging Cell. 2021 Jun 15;20(7):e13382. doi: 10.1111/acel.13382 (PMC8282275; doi:10.1111/acel.13382)

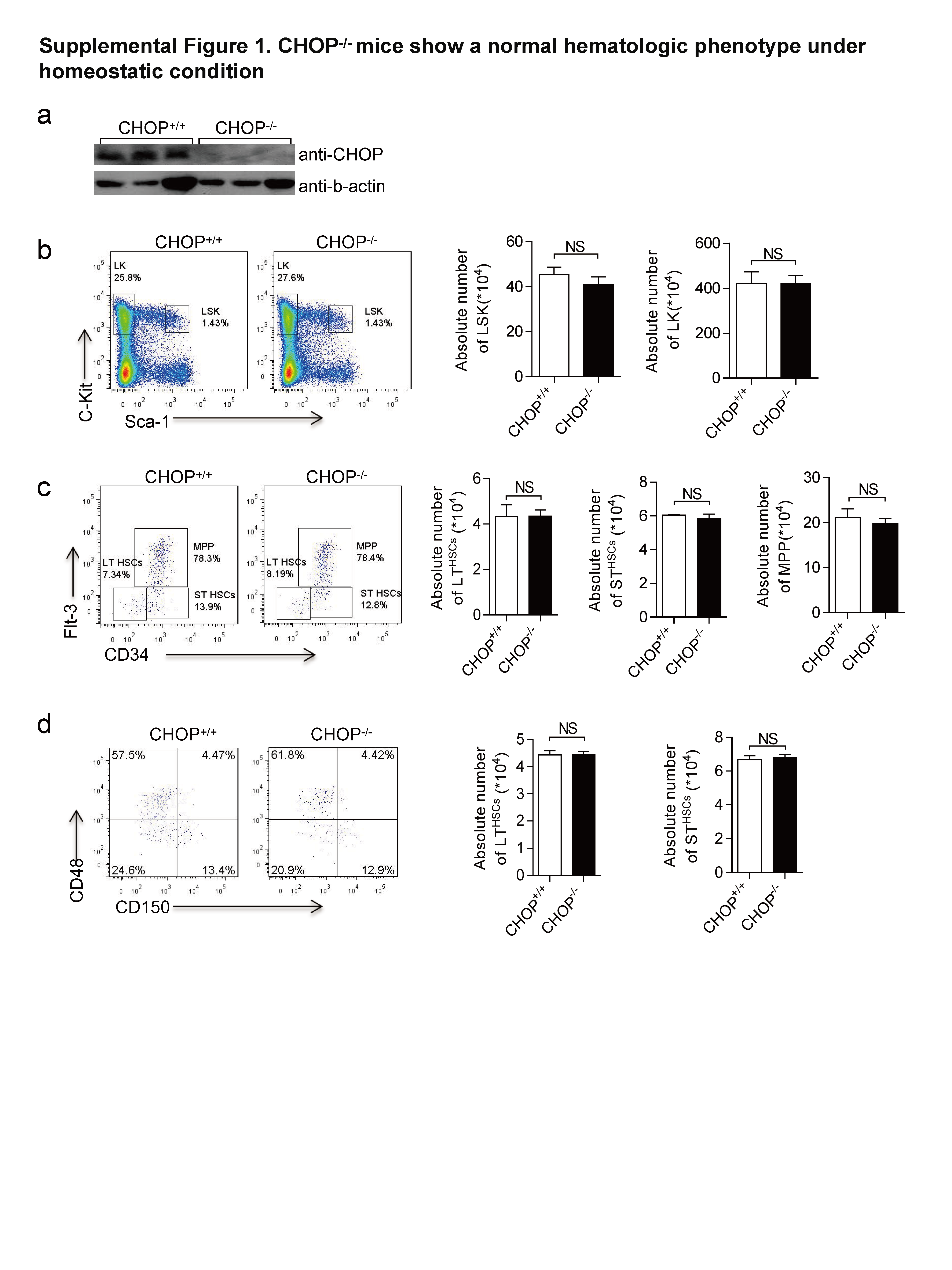

Supplement: Supplementary file 1 — Figure S1 [file ACEL-20-e13382-s001.zip › acel13382-sup-0001-FigS1-1.tif]

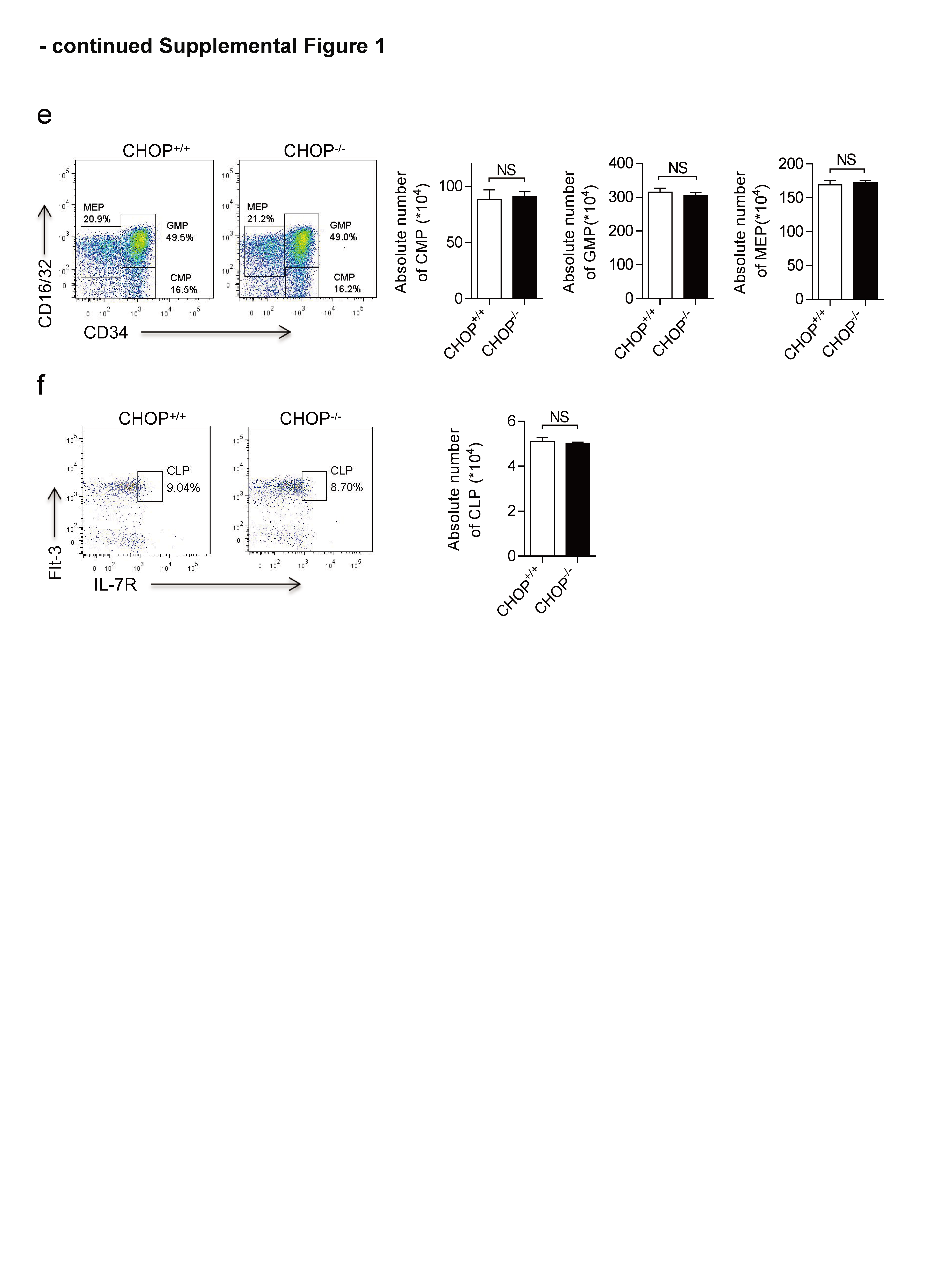

Supplement: Supplementary file 1 — Figure S1 [file ACEL-20-e13382-s001.zip › acel13382-sup-0002-FigS1-2.tif]

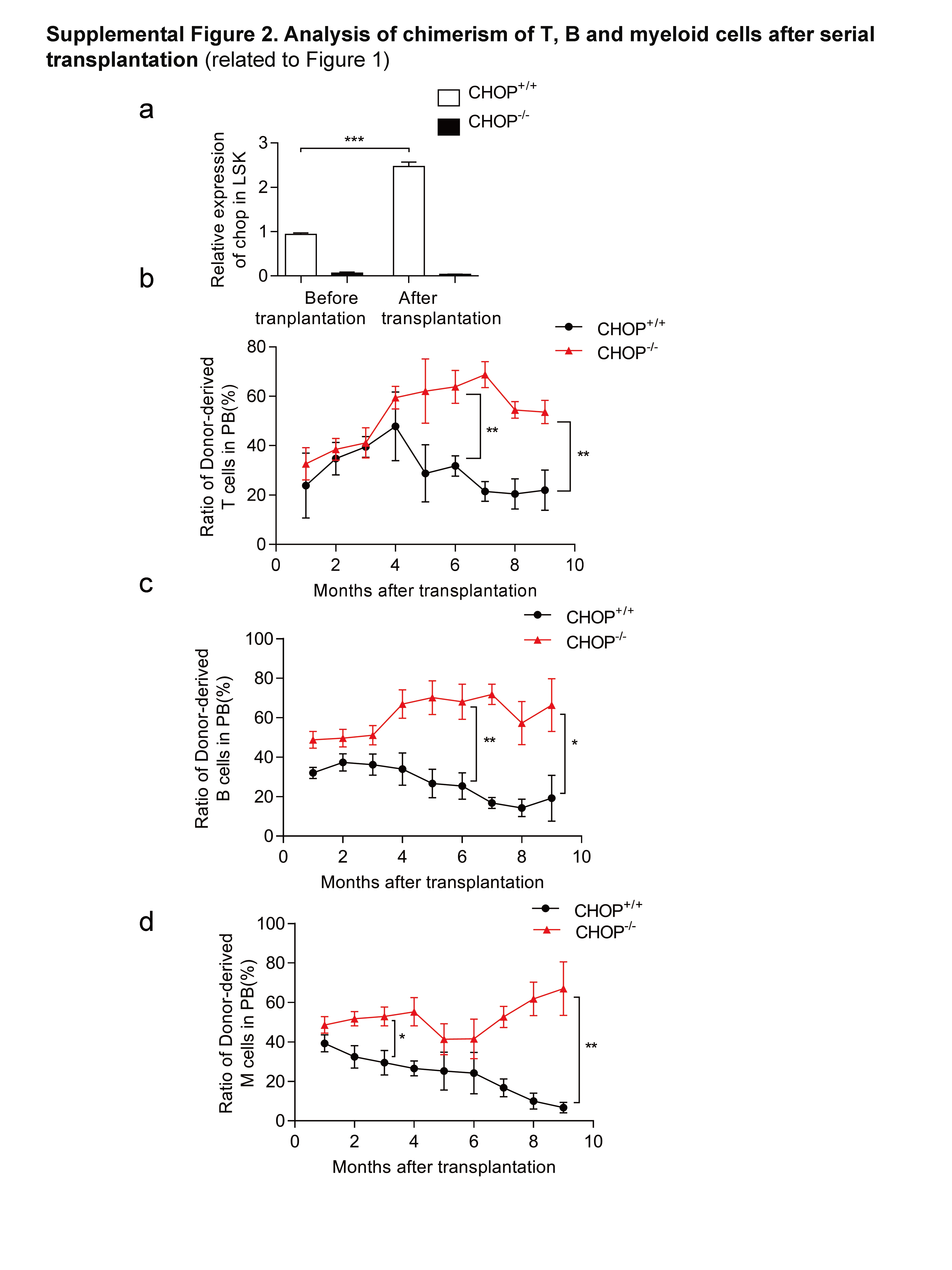

Supplement: Supplementary file 2 — Figure S2 [file ACEL-20-e13382-s002.zip › acel13382-sup-0003-FigS2-1.tif]

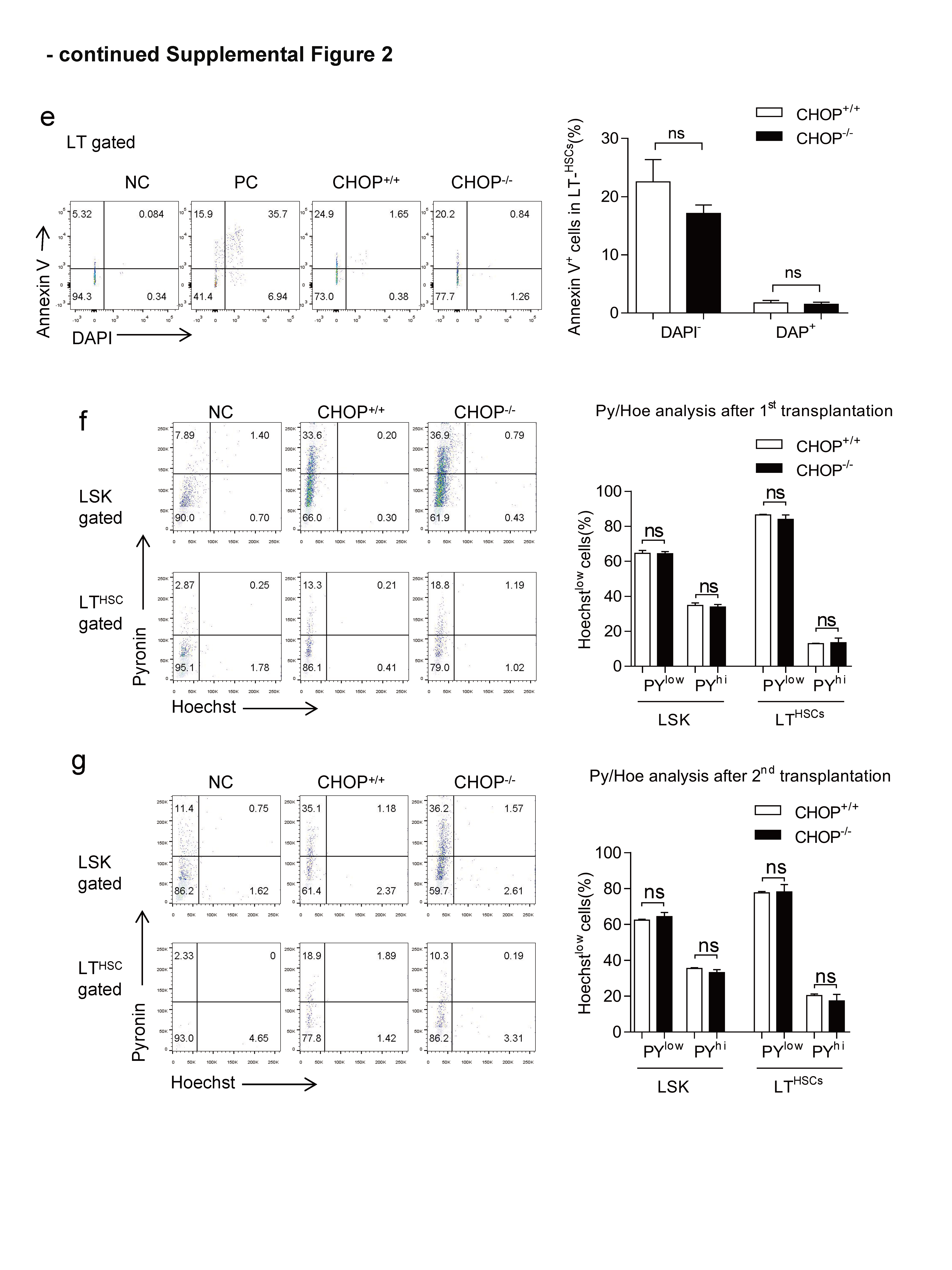

Supplement: Supplementary file 2 — Figure S2 [file ACEL-20-e13382-s002.zip › acel13382-sup-0004-FigS2-2.tif]

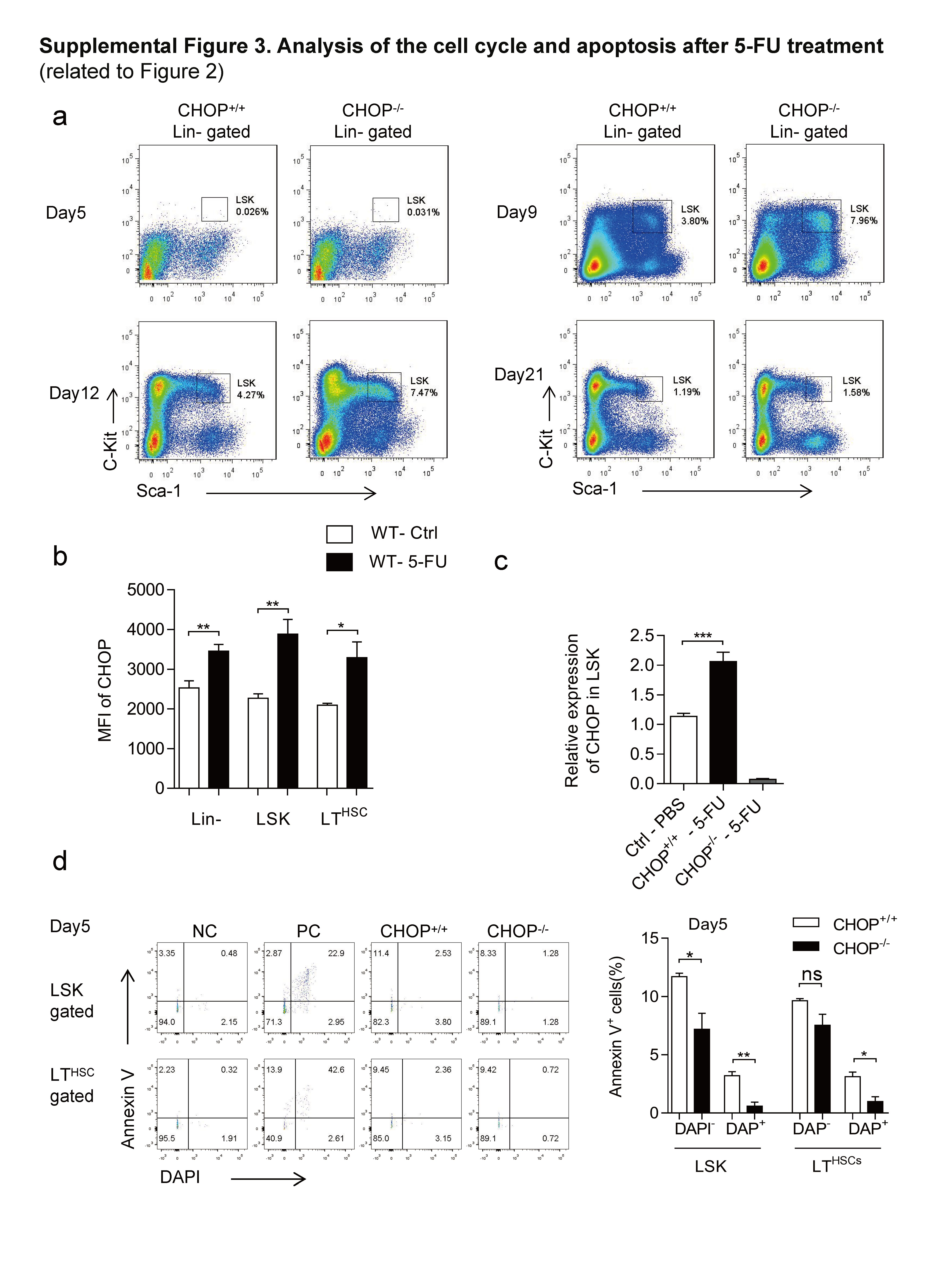

Supplement: Supplementary file 3 — Figure S3 [file ACEL-20-e13382-s005.zip › acel13382-sup-0005-FigS3-1.tif]

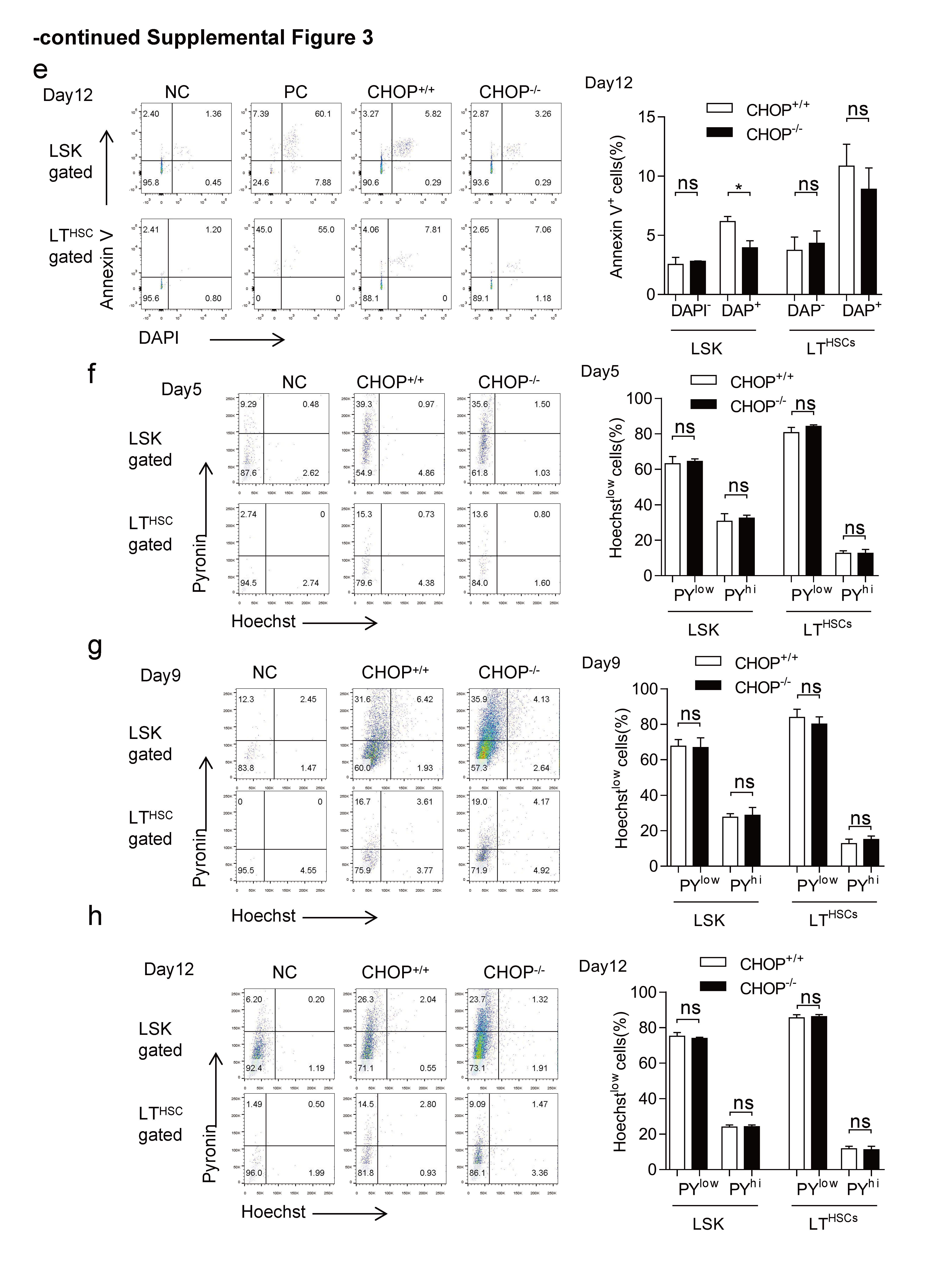

Supplement: Supplementary file 3 — Figure S3 [file ACEL-20-e13382-s005.zip › acel13382-sup-0006-FigS3-2.tif]

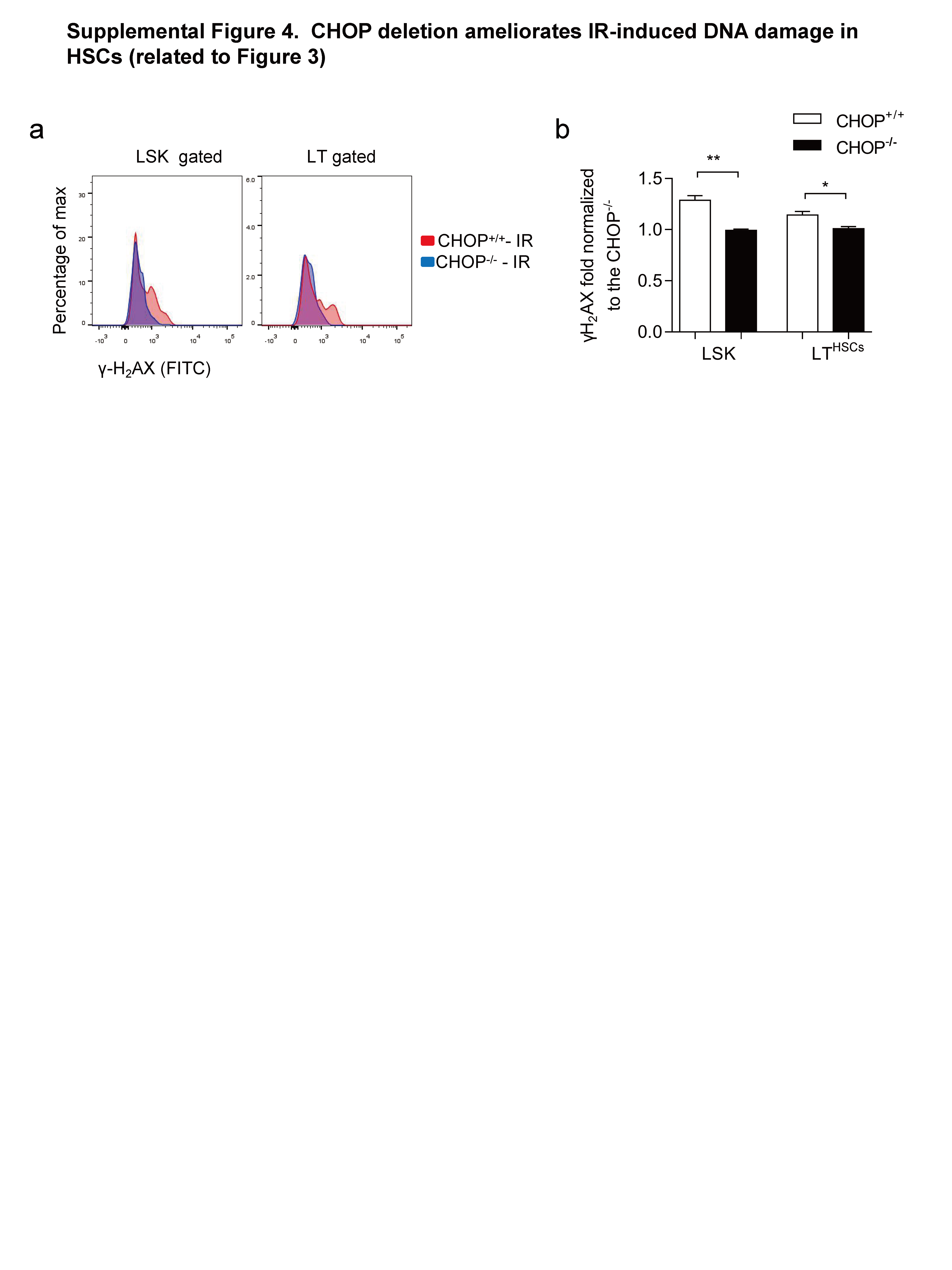

Supplement: Supplementary file 4 — Figure S4 [file ACEL-20-e13382-s006.tif]

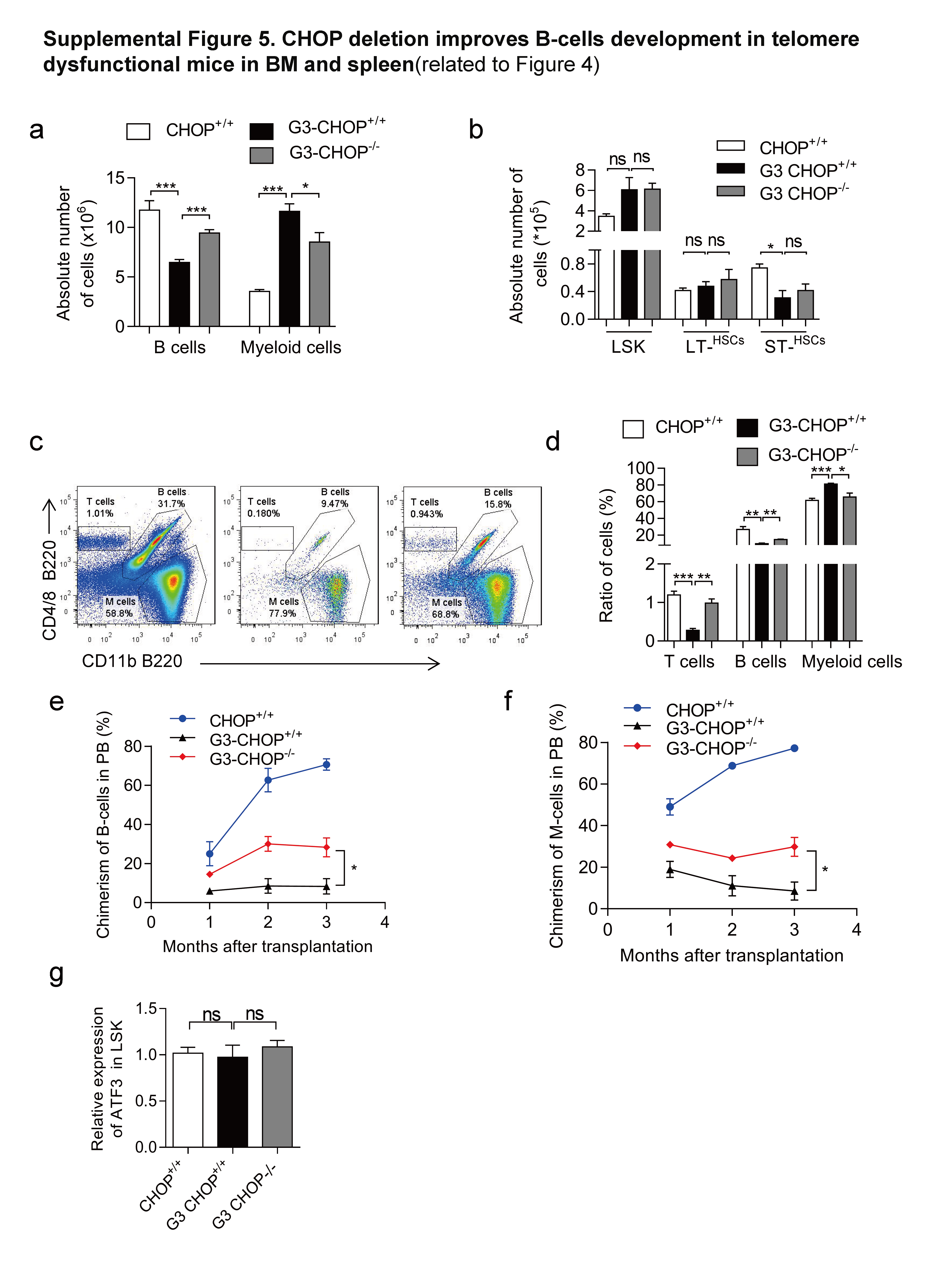

Supplement: Supplementary file 5 — Figure S5 [file ACEL-20-e13382-s007.tif]

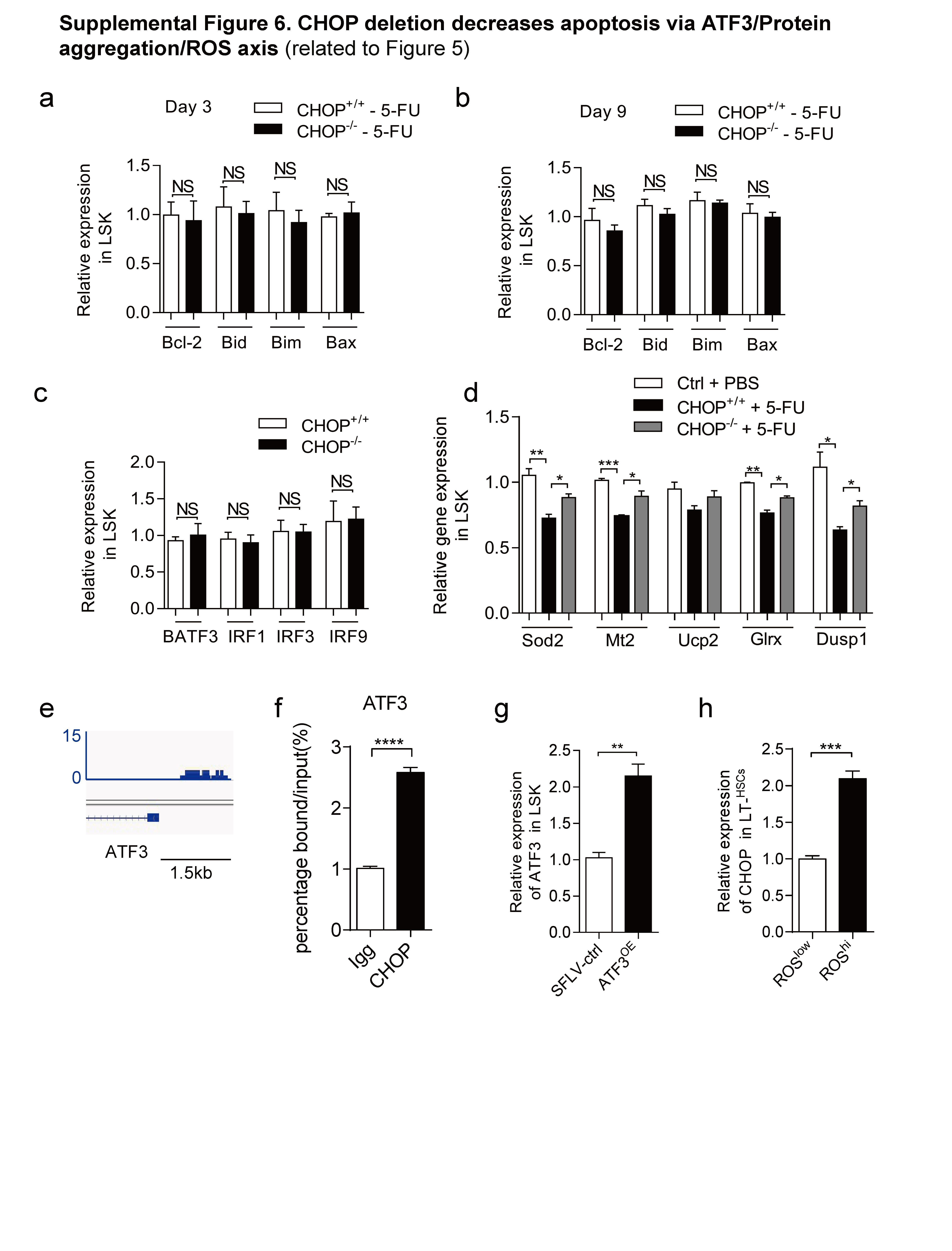

Supplement: Supplementary file 6 — Figure S6 [file ACEL-20-e13382-s003.tif]
