# Supplementary material for: C/EBP homologous protein deficiency enhances hematopoietic stem cell function via reducing ATF3/ROS‐induced cell apoptosis
Source: Aging Cell. 2021 Jun 15;20(7):e13382. doi: 10.1111/acel.13382 (PMC8282275; doi:10.1111/acel.13382)
Supplement: Supplementary file 7 — Supplementary Material [file ACEL-20-e13382-s004.docx]

**Supplemental Figure 1. CHOP^-/-^ mice show a normal hematologic phenotype under homeostatic condition**

(a) Western blot analysis of WT and CHOP^-/-^ BM cells by using anti-CHOP antibody, beta-actin was used as sample loading control. (b-f) The absolute number of LSK (Lin^-^c-Kit^+^Sca-1^+^) cells, LK (Lin^-^c-Kit^+^Sca-1^-^, LKS^-^), LT-HSCs (CD34^-^Flt3^-^Lin^-^c-Kit^+^Sca-1^+^), ST-HSCs (CD34^+^Flt3^-^Lin^-^c-Kit^+^Sca-1^+^), MPPs (CD34^+^Flt3^+^Lin^-^c-Kit^+^Sca-1^+^), LT-HSCs (CD48^-^ CD150^+^Flt3^-^Lin^-^c-Kit^+^Sca-1^+^), ST-HSCs (CD48^-^CD150^-^Flt3^-^Lin^-^c-Kit^+^ Sca-1^+^), common myeloid progenitors (CD34^+^CD16/32^-^LKS^-^), GMPs (CD34^+^CD16/32^+^LKS^-^), megakaryocyte/erythroid progenitors (CD34^-^CD16/32^-^LKS^-^) and common lymphoid progenitors (Flt3^+^IL-7R^+^Lin^-^c-Kit^low^Sca-1^low^) in young mice was determined by FACS (n = 3-4 per group). *p<0.05; NS, not significant

**Supplemental Figure 2. Analysis of chimerism of T, B and myeloid cells after serial transplantation** (related to Figure 1)

(a) CHOP mRNA expression in LSK from CHOP^+/+^ / CHOP^-/-^ mice before and after transplantation was measured via real-time PCR. The relative expression was normalized to β-actin for statistical analysis (n = 3 per group). (b-d) Three-round serial transplantation was conducted using 4000 purified LSK cells along with 1*10^6^ fresh competitors each time. Chimerism of T, B and M cells in PB is shown at the indicated time points after transplantation. (e) The apoptosis was detected with Annexin V/DAPI staining in LT-HSCs after first transplantation 10days (n=3-4 per group). (f-g) The cell cycle analysis was detected with PY/Hoechst staining in LSK cells and LT-HSCs. The percentage of the cell cycle distribution is shown (n=3 per group). *p<0.05;**p<0.01; NS, not significant.

**Supplemental Figure 3. Analysis of the cell cycle before and after 5-FU treatment** (related to Figure 2)

(a) The representative fluorescence-activated cell sorting (FACS) plots of the LSK population are shown at the indicated time points after 5-FU treatment. The frequency of LSK (Lin^-^Sca-1^+^c-kit^+^) cells in Lin- cells between CHOP^+/+^ and CHOP^-/-^ mice are shown. (b) Change of CHOP median fluorescence intensity measured by FACS in LSK cells at day 9 mice after 5-FU treatment (n = 4 per group). (c) CHOP mRNA expression in LSK from CHOP^+/+^ and CHOP^-/-^ mice at day 9 after 5-FU injection was measured via real-time PCR. The relative expression was normalized to β-actin for statistical analysis (n = 3-4 per group). (d-e) The apoptosis analysis was detected with Annexin V/DAPI staning in LSK cells and LT-HSCs after 5-FU treatment 5 and 12 days(n=3 per group). (f-h) The cell cycle analysis was detected with PY/Hoechst staining in LSK cells and LT-HSCs after 5-FU treatment 5,9 and 12 days. The percentage of the cell cycle distribution is shown (n=3 per group).*p<0.05;**p<0.01; NS, not significant.

**Supplemental Figure 4. CHOP Deletion Ameliorates IR-induced DNA Damage in HSCs (**related to Figure 3**)**

(a-b) The representative FACS plots and quantification of Gamma-H2AX immunofluorescence staining after bone marrow transplantation after 4.5-Gy IR 2 hours are shown(n=3 per group).

**Supplemental Figure 5. CHOP deletion improves B-cells development in telomere dysfunctional mice in BM and spleen** (related to Figure 4)

(a) Absolute number of B (B220^+^) cells and M (CD11b^+^) cells in the spleen compared with Terc^-/-^ mice. CHOP deletion improves B-cells development and decreases myeloid proliferation in Terc-/- mice (n = 3-5 per group). (b) Absolute number of LSK cells, LT-HSCs and ST-HSCs in the bone marrow in Terc^+/+^, Terc^-/-^ CHOP^+/+^and Terc^-/-^ CHOP^-/-^ mice (n = 3-4per group). (c-d) Percentages of T, B and myeloid cells in BM, 12 weeks after transplantation. Representative FACS plots are shown on the left(C) and statistical data is shown on the right (D) (n = 3-4per group). (e-f) Chimerism of B and M cells in PB was shown at the indicated time points after transplantation. (g) ATF3 mRNA expression in LSK from CHOP^+/+^, G3 Terc^-/-^ and G3 Terc^-/-^ CHOP^-/-^ mice was measured via real-time PCR. The relative expression was normalized to β-actin for statistical analysis (n = 3per group). *p<0.05; **p<0.01; NS, not significant

**Supplemental Figure 6. CHOP deletion decreases apoptosis via ATF3/Protein aggregation/ROS axis** (related to Figure 5)

1. b) The mRNA expression of apoptosis-related genes Bcl-2, Bid, Bim and Bax in LSK from CHOP+/+ and CHOP-/- mice at day 3 and 9 after 5-FU administration was measured via real-time PCR. The relative expression was normalized to β-actin for statistical analysis (n = 3 per group). (c) The mRNA expression of BATF3, IRF1, IRF3 and IRF9 in LSK from CHOP^+/+^ and CHOP^-/-^ mice was measured via real-time PCR. The relative expression was normalized to β-actin for statistical analysis (n = 3-4 per group).(d) Expression profiles of anti-oxidant genes in response to ER stress. LSK cells were sorted after 9days of 5-FU administration(n = 3 per group). (e) Track image shows the indicated ChIP-seq experiments on the ATF3 locus. (f) Real-time PCR analysis of CHOP chromatin immunoprecipitated DNA. For Q-pcr primer, amplifications on chromatin before immunoprecipitation and chromatin immunoprecipitated with preimmune serum were performed as input and negative control, respectively. The value of bound DNA relative to input in percentage is shown (n = 3 per group). (g) The mRNA expression of ATF3 from SFLV-ctrl and ATF3^OE^ GFP^+^ cells (n=3 per group). (h) The mRNA expression of CHOP in ROS^low^ and ROS^high^ LT-HSCs (n=3 per group). *p<0.05; **p<0.01; NS, not significant
